# Enhanced tumor immunotherapy by polyfunctional CD19-CAR T cells engineered to secrete anti-CD47 single-chain variable fragment

**DOI:** 10.7150/ijbs.86632

**Published:** 2023-09-18

**Authors:** Yingqi Qiu, Peiyun Liao, Hao Wang, Jianyu Chen, Yuxing Hu, Rong Hu, Honghao Zhang, Zhongwei Li, Manxiong Cao, Yulu Yang, Meifang Li, Xiaoling Xie, Yuhua Li

**Affiliations:** 1 Department of Hematology, Zhujiang Hospital, Southern Medical University, Guangzhou, Guangdong, 510280, P. R. China.; 2 Bioland Laboratory (Guangzhou Regenerative Medicine and Health Guangdong Laboratory), Guangzhou, Guangdong, 510005, P. R. China.

**Keywords:** Chimeric antigen receptor T cell, Non-Hodgkin's lymphoma, CD47, T cell polyfunctionality, macrophage

## Abstract

A high recurrence rate of non-Hodgkin's lymphoma (NHL) following chimeric antigen receptor T (CAR T) cell treatment remains a bottleneck, and immunosuppressive tumor microenvironment (TME) compromising CAR T cell efficacy in NHL is the primary cause of relapse. Accordingly, modifying the structure of CAR T cells to attenuate the inhibitory effect of TME thus reducing recurrence rate is a valuable research topic. CD47 has been proved to be a promising therapeutic target and is crucial in regulating macrophage function. Herein, we engineered CD19-CAR T cells to secrete an anti-CD47 single-chain variable fragment (scFv) and validated their function in enhancing antitumor efficacy, regulating T cells differentiation, modifying phagocytosis and polarization of macrophages by* in vitro* and *in vivo* researches. The efficacy was analogous or preferable to the combination of CAR T cells and CD47 antibody. Of note, anti-CD47 scFv secreting CAR T cells exert a more potent immune response following specific antigen stimulation compared with parental CAR T cells, characterized by more efficient degranulation and cytokine production with polyfunctionality. Furthermore, locally delivering anti-CD47 by CAR T cells potentially limits toxicities relevant to systemic antibody treatment. Collectively, our research provides a more effective and safer CAR T cell transformation method for enhancing tumor immunotherapy.

## Introduction

Non-Hodgkin's lymphoma (NHL) is a group of heterogeneous tumors with monoclonal amplification of malignant lymphocytes 1. Approximately 20%-40% of patients experience disease recurrence or progress to refractory NHL after conventional chemotherapy, with a 5-year overall survival rate of only 38.3% 2. Consequently, there is an urgent need to explore new therapeutics to improve the prognosis of NHL patients. As a novel targeted immunotherapy, chimeric antigen receptor T (CAR T) cells have exerted excellent efficacy in hematologic malignancies 3, which can specifically recognize tumor cells expressing target antigens without MHC molecule processing and presentation, rapidly initiating killing function and achieving accurate tumor clearance 4, 5. Presently, CAR T cells targeting CD19 are most widely used in the treatment of relapsed or refractory (R/R) leukemia and lymphoma patients. However, the challenge lies in the high risk of recurrence despite the initial high remission rate. Results from multiple clinical studies have shown that while 50% of R/R NHL patients could achieve complete remission applying CD19-CAR T cells, approximately 55% of them experience relapse within one year 6, 7. The most likely reason for this phenomenon is that there are numerous fibrous stroma and immunosuppressive cells exist in the tumor microenvironment (TME) of lymphoma. The physical barrier and immune blockade constituted by them protect tumor cells against attack from immune cells, which are instrumental in promoting biological behaviors such as tumor proliferation, dissemination and immune evasion 8. Moreover, multiple inhibitory signals in TME inhibit the cytotoxicity and persistence of CAR T cells, leading to the so-called depletion state 9. Therefore, to alleviate the inhibitory effect of immunosuppressive TME on CAR T cells, it is necessary to further upgrade the structure of existing CD19-CAR T cells to obtain better efficacy.

Tumor-associated macrophages (TAMs) play a key role in the immunosuppressive cell and cytokine network of TME and involve in inducing immune escape of tumor cells by multiple means such as mediating tumor antigen recognition disorders 10, primarily through CD47/SIRPα axis 11. Normal cells express CD47 to label themselves, releasing a “don't eat me” signal to protect themselves from macrophage-mediated phagocytosis 12. Nevertheless, CD47 is also highly expressed in various tumors 13, through which cancer cells release anti-phagocytic signals to masquerade as normal cells, thereby causing immune escape and promoting tumor progression 14. Consequently, CD47 is a potential therapeutic target for relieving TME immunosuppression. Currently, several clinical studies of CD47 monoclonal antibodies are under way, and have achieved considerable antitumor effects, but the resulting cytotoxic reactions such as hemocytopenia cannot be ignored 15, 16.

To address these limitations, we developed a novel CD19-CAR T cell secreting anti-CD47 single-chain variable fragment (scFv) and validated its efficacy, safety, along with immunomodulatory effects on TME by *in vitro* and *in vivo* experiments. Genetically engineered CD19-CAR T cells to secrete anti-CD47 scFv effectively enhance the polyfunctionality and tumor killing function of CAR T cells, uplifting their persistence and immune activity and facilitating their differentiation into naive phenotypes. In addition, the CAR T cells also acts as a medium to achieve local delivery of anti-CD47 antibodies, reduce the impact on normal cells, and block the anti-phagocytosis effect of tumor cells, thus ameliorating the immunosuppressive TME, diminishing tumor immune evasion.

## Materials and methods

### Patient specimens

The bone marrow (BM) samples of NHL patients were obtained from the Hematology Department of Zhujiang Hospital, Guangzhou, China. Peripheral blood (PB) samples from healthy donors were also collected as control. The patient information of specimens used in this study can be seen in **Table S1**.

### Cell culture

Human NHL cell lines (Raji, Jeko1, Farage, Daudi, Ramos, Namalwa), lymphoblastic leukemia cell line Nalm6, and 293T were maintained in Hematological Laboratory of Zhujiang Hospital (Southern Medical University, China) and genotyped to verify their identity. All cell lines were acquired from the American Type Culture Collection or Deutsche SammLung von Mikroorganismen und Zellkulturen. Cell lines were identified by short tandem repeat (STR) profiling and confirmed to be mycoplasma-free immediately before use. Cells over passage 15 were not used. 293T cells were maintained in high-glucose DMEM (BIOLOGICAL INDUSTRIES, Israel) supplemented with 10% fetal bovine serum (FBS, Moregate, Australia). Other cell lines were cultured in RPMI-1640 media (VivaCell, Shanghai, China) supplemented with 10% FBS. Raji cells transduced with luciferase were used in *in vivo* experiments as indicated.

### Lentiviral CAR vectors design and construction

The CD19-CAR composed of a CD8α signal peptide, a human anti-CD19 scFv, a CD8α hinge, a CD8α transmembrane, a 4-1BB intracellular costimulatory domain and an intracellular CD3ζ domain, which was cloned into the pCDH-EF1-MCS lentiviral vector. The vector encoding CD19-s47-CAR was then generated by linking CD19-CAR and HA-tagged human anti-CD47 scFv with a “self-cleaving” T2A peptide. Corresponding CAR sequences can be found in **Table S2**. Subsequently, the above CAR-expression plasmids were respectively mixed with packaging plasmids pMD2.G as well as psPAX2, and transfected into 293T cells with Lipofectamine 3000 (Thermo Fisher). The culture supernatants were then harvested and concentrated for later use.

### T cell isolation and modification

Peripheral blood mononuclear cells (PBMCs) were isolated from fresh peripheral blood of healthy donors by Ficoll-Paque density gradient medium (TBDSCIENCE, Tianjin, China), and then T cells were separated by CD3 positive selection kit (STEMCELL, Canada) in accordance with the manufacturer instructions. T cells were seeded at 1 × 10^6^/mL in X-vivo serum free medium (LONZA, Basel, Switzerland) supplemented with IL-2 (500 IU/mL, Sino Biological, Beijing, China) and activated with Human CD3/CD28 T Cell Activator (STEMCELL) for 24 h. After that, CD19-CAR T and CD19-s47-CAR T cells were obtained by transfecting with the aforementioned viruses at an MOI of 10 in the presence of polybrene (10 µg/mL), and the transfection rate of CAR T cells was detected 3 days after transfection by anti-CAR antibody CARGREEN (Yake, Shanghai, China). About 7 to 14 days after transfection, CAR T cells were collected for follow-up experiments.

### Western blot analysis

For supernatant protein extraction, proteins were precipitated using a high-performance protein precipitation kit (Invent, Beijing, China). For total protein extraction, cells were solubilized in ice-cold RIPA buffer (KeyGEN, Jiangsu, China) containing protease and phosphatase inhibitor cocktail (Solarbio, Beijing, China). Protein concentrations were subsequently quantified by BCA Protein Quantitative Kit, resolved on SDS-PAGE gels (Beyotime, Shanghai, China), and transferred to PVDF membranes (PALL, New York, USA). Membranes were blocked in 5% non-fat dried milk for 2 h at room temperature, then incubated overnight at 4°C with primary antibodies. After washed three times in PBST, membranes were incubated for 1 h at room temperature with HRP-conjugated secondary antibodies. Finally, blots were infiltrated in FDbio-Dura ECL luminescent solution (Fdbio science, Hangzhou, China) and photographed using BioRad Imaging System.

### Determination of secreted anti-CD47 antibody concentration

Culture supernatants of different T cells were collected and enriched for secreted anti-CD47 antibody by Anti-HA Tag Magnetic Beads Immunoprecipitation Kit (Sino Biological). Supernatants were incubated with HA-tagged beads for 30 min at 37°C with rotating mixing, placed in a magnetic stand to adsorb beads, and then eluted with neutral eluent. Subsequently, the concentration of anti-CD47 antibody in the eluate was determined by BCA quantification (Keygen).

### Flow cytometry analysis

For extracellular staining of* in vitro* experiments, cells were harvested and washed with PBS buffer, followed by staining with various antibodies diluted in Flow Cytometry Staining Buffer (FACS buffer) at room temperature for 30 min. After washing the cells with PBS and centrifuging to discard the supernatant, the cells were resuspended in PBS and analyzed on CytoFlex (BECKMAN, Frankfurt, Germany). For flow cytometry detection of *in vivo* experiments, mouse samples were prepared as single cell suspension and red cells were lysed by red cell lysis buffer (BOSTER, Wuhan, China) before staining. Following blocking non-specific binding with Human TruStain FcX™ (Fc Receptor Blocking Solution, Biolegend, Santiago, USA), subsequent experiments were performed in line with the staining steps described above. All antibodies used in this work can be found in **Table S3**. CytExpert and FlowJo software were utilized to analyze and plot the data.

### CD107a degranulation assay

To determine the degranulation function of different T cells in response to antigen stimulation, 5 × 10^4^ T cells were plated with 5 × 10^4^ Raji in a 96-well plate with U-shaped bottom. Monensin (Biolegend, San Diego, USA) and an anti-CD107a antibody (Biolegend) were subsequently added, and the plate was cultured at 37°C for 4 h. Then, cells were harvested and stained with surface markers (CD3, CD8 and CAR), followed by flow cytometry detection on CytoFlex.

### Intracellular staining

T cells and Raji cells were plated at an E:T ratio of 1:1 (1 × 10^5^ effectors to 1 × 10^5^ targets) in a 96-well plate with U-shaped bottom. Cytokine exocytosis of T cells was blocked by supplementing protein transport inhibitor brefeldin-A (BFA, BD Biosciences, New York, USA) to the wells. The plate was incubated at 37˚C for 4 h. After that, cells were collected and then stained with surface markers (CD3, CD8 and CAR), followed by fixation and permeabilization utilizing a Fix and Perm kit (Biolegend). T cells were then stained with anti-IL-2, TNF-α, or/and IFN-γ flow antibodies at 4°C for 30 min in the dark. Stained cells were washed and resuspended in chilled PBS for flow cytometry.

### Polyfunctionality profile measurement

T cells were incubated with Farage cells in the presence of BFA at 37˚C for 4 h, and then stained with surface markers at 4°C for 30 minutes, followed by fixation and permeabilization. Next, anti-IL-2, TNF-α, and IFN-γ antibodies were added and cells were incubated at 4°C for 30 min to conduct polyfunctionality analysis. After multiple intracellular cytokine staining, cells were detected on CytoFlex and analyzed by FCS Express 7 software. For each sample, 10,000 cells were subjected to tSNE distribution and labeled according to polyfunctionality. Exported results were further adjusted and encapsulated as pie charts.

### ELISA

After incubating 1 × 10^5^ T cells with 1 × 10^5^ Raji in a 96-well plate for 5, 24 and 48 hours, the culture supernatant was collected and subsequently analyzed for cytokine production. The expression levels of cytokines (IL-2, IFN-γ and TNF-α) in T cell culture supernatant were determined by ELISA. The assay kits were purchased from Biolegend and used in accordance with the manufacturer instructions.

### LDH cytotoxicity assay

Cytotoxicity experiments were performed on T cells on day 7 after transfection. NHL cell lines such as Raji, Jeko1, and Farage were also collected and adjusted to the density of 1.6 × 10^5^/mL, then inoculated into round-bottom 96-well plates at 50 ul per well. T cells were added into the plate according to the E:T ratios of 20:1, 10:1, 5:1, and 2.5:1, respectively, and the plate was incubated at 37˚C with 5% CO_2_ for 4 h. Cytolytic activity was measured using a standard lactate dehydrogenase (LDH)-release assay (Promega, Wisconsin, USA), and the specific operation was performed according to the kit instructions. The percentage of specific lysis was determined according to the following formula: [(experimental LDH release-spontaneous LDH release)/(maximum LDH release-spontaneous LDH release)] × 100.

### T-cell expansion and cell division assay

For proliferation assays, T cells were labeled with CellTrace Far Red (1 μmol/L, Invitrogen, California, USA) for 5 min at 37˚C. The reaction was terminated by adding 1 mL RPMI-1640 complete medium and the cells were washed three times. Then T cells were incubated with irradiated Raji cells at a 1:1 ratio for 4 days without exogenous cytokines. Next, cells were stained with anti-CD3, CD8 antibodies. Dilution of Cell Trace Far Red were examined via CytoFlex and analyzed by FlowJo software.

### Apoptosis analysis

NHL cell lines such as Raji, Jeko1, and Farage were labeled with CFSE and plated with T cells into a round-bottom 96-well plate at the E:T ratios of 20:1, 10:1, 5:1, and 2.5:1, at 37˚C with 5% CO2 for 4 h. After that, cells were harvested and stained with 7-amino-actinomycin D (7-AAD) Apoptosis Detection Kit (KEYGEN, Nanjing, China) using the buffer provided according to the manufacture's guidelines, followed by flow cytometry detection on CytoFlex.

### Quantitative real-time PCR (qPCR) analysis

Total RNA was extracted from samples using TRIzol reagent (Invitrogen) and reverse-transcribed into cDNA applying HiScript III RT SuperMix for qPCR kit (Vazyme, Nanjing, China). Specific targets were amplified from the cDNA using the SYBR Green Dye detection system (Takara, Japan) on a Real-Time PCR System (BIO-RAD). The 2^-ΔΔCt^ method was utilized to normalize the relative expression levels of the target genes with GAPDH as internal control. All primer sequences used in this work were listed in **Table S4**.

### Generation of macrophages

Monocytes in PBMC isolated from fresh peripheral blood of healthy donors were obtained using CD14 positive selection kit (STEMCELL) in accordance with the manufacturer instructions. Human macrophages (M0) were generated by culturing monocytes with RPMI-1640 supplemented with 10% FBS and human M-CSF (20 ng/mL, PEPROTECH, New Jersey, USA) for 6 days at 37°C. Meanwhile, the culture system was renovated with fresh cytokine-containing medium on days 3 and 5.

### *In vitro* phagocytosis assay

For the *in vitro* phagocytosis analysis, macrophages were cultured with carboxyfluorescein succinimidyl ester (CFSE)-tagged tumor cells in the presence of different T cells at a ratio of 2:1:1 (macrophages to tumor cells to T cells) at 37°C for 3 h. After incubation, cells were stained with anti-CD14 antibodies to identify macrophages, and the percentage of CFSE^+^ macrophages was characterized as phagocytosis rate.

### Animal models

B-NDG mice used for *in vivo* experiments were obtained from BIOCYTOGEN (China). Mice were injected with 2 × 10^5^ luciferase-expression Raji cells via tail vein to establish NHL mouse models. Tumor progression was monitored by IVIS Spectrum (PerkinElmer). Mice successfully established as lymphoma models were randomly divided into 5 groups of 8 mice each. At day 7, 1 × 10^6^ T cells were adoptively transferred through intravenous injection. One day after T cell infusion, Hu5F9 was administered via intraperitoneal injection (*i.p.*) at a dose of 1 mg/kg on the indicated date. Five days post treatment, orbital blood collection was conducted for blood routine test. Mice were observed daily for diet, status as well as body weight, and tumor distribution was monitored weekly using an IVIS Spectrum. Animals were euthanized if they began to show signs of immobility, curling, refusal to eat, fur folds, or self-abuse.

The patient-derived xenograft (PDX) models were created as previously described 17, using viable cryopreserved NHL specimens banked at the Hematology Department of Zhujiang Hospital in accordance with research protocols approved by the institutional review board. Briefly, primary NHL cells from bone marrow were intravenously injected into B-NDG mice. The bone marrow cells and splenocytes harvested from successfully engrafted xenografts were reinjected to create secondary (P2) and tertiary (P3) xenografts for the treatment trials. Engraftment was verified by flow cytometric analysis of peripheral blood using antibodies against human CD19.

For humanized PBMC models, 2 × 10^6^ human PBMCs were injected intravenously into each B-NDG mouse. Peripheral blood from all mice was monitored for human immune system reconstruction (hCD45^+^). Mice showed peripheral chimerism by 6 weeks. Successfully reconstructed models were subcutaneously (*s.c.*) injected with 2 × 10^6^ above splenocytes from P4 PDX model into the right flank of mice to construct PBMCs-PDX models. After developing visible subcutaneous tumors (day 10), subsequent T cell adoptive therapy and antibody treatment were performed. Tumor volumes were recorded every five days according to the formula: (length × width^2^) / 2. On day 30, tumor tissues were removed from all mice following euthanasia. Single cell suspensions were prepared from their liver, femur, and spleen to inspect T cell persistence, tumor infiltration and immune cell proportions in different groups.

### H&E staining and immunohistochemistry

Major organ samples (femur, liver, lung, and spleen) from NHL mice were collected and fixed in 4% paraformaldehyde. After paraffin embedding, slices were cut into 4 µm and subjected to subsequent H&E staining or IHC staining. IHC for hCD47 of clinical patient tissue samples (**Table S5**) or hCD19 of mice samples were performed on slices following a series of operations such as dewaxing, hydration, antigen repair, sealing, etc. After staining, the sections were observed and analyzed with a Leica upright brightfield microscope.

### Statistics

Data were analyzed using GraphPad Prism 8 software and results were presented as mean ± SD. Unpaired Student's t-test was applied to assess the statistical significance between two independent groups. For multiple comparisons, One-way ANOVA with Turkey's multiple comparison test was applied for statistical analyses. Survival curves were calculated by the Kaplan-Meier method and compared by the log-rank test. Statistical significance was determined as * *P* < 0.05, ** *P* < 0.01, *** *P* < 0.001, ns (no significant difference).

## Results

### NHL-polarized macrophages exhibit M2 phenotype and inhibit CAR T cell expansion

**Figure 1:**
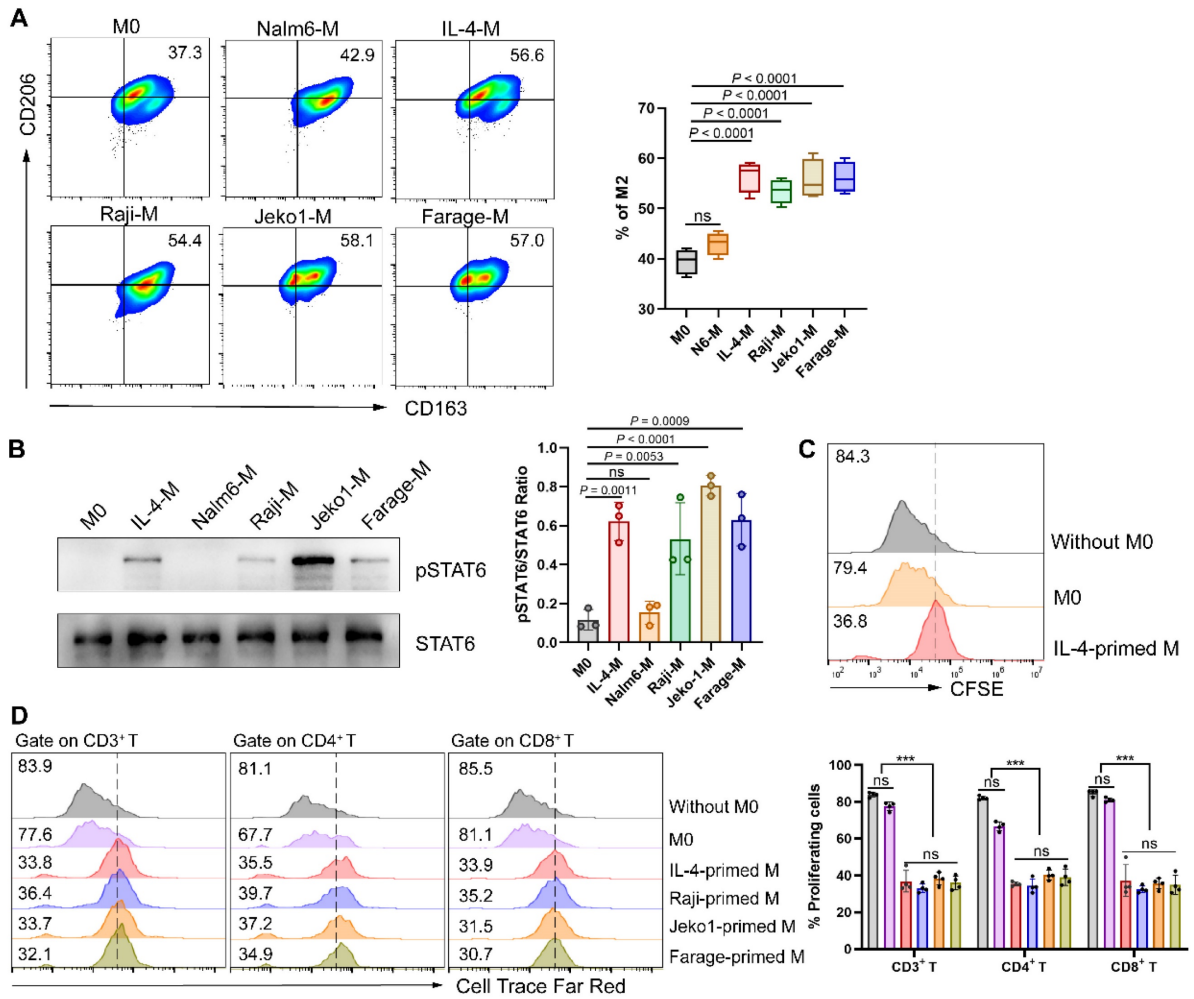
** NHL cells promote macrophage polarization to M2 thus inhibiting CAR T cell proliferation. (A)** Mononuclear cells obtained from fresh PBMCs were induced to M0 by 1640 medium containing 10% FBS and 20 ng/mL M-CSF after 6 days of culture, followed by culturing respectively with a control B-cell acute lymphoblastic leukemia cell line Nalm6, IL-4 (M2 positive control), or NHL cell lines (Raji, Jeko1 and Farage) for 24 h. Then flow cytometry analysis gating on CD14^+^ CD16^+^ macrophages and quantification of M2 phenotypes (CD163^+^ CD206^+^) were conducted (n = 4). **(B)** Western blot analysis showing STAT6 phosphorylation of macrophages under different culture conditions recapitulated in A (left). Densitometric analysis was conducted utilizing Image J software. Statistical analysis plot of the triplicate experiments on the ratio of the IntDen value of pSTAT6 to STAT6 can be found in on the right side. All samples were derived from the same experiment and that gels were processed in parallel. **(C)** IL-4-polarized macrophages abated CD19-CAR T cell proliferation in response to irradiated CD19^+^ Nalm6 cells, as shown by a 72 h CFSE dilution assay. **(D)** NHL-polarized macrophages attenuated Nalm6-stimulated proliferation of CD19-CAR T cells as IL-4-polarized macrophages. Pretreatment of macrophages with conditions in **A** and then co-cultured with Cell Trace Far Red-labeled CAR T cells for a 72 h proliferation experiments. Representative flow cytometry images (left), corresponding statistical chart (right) are shown (n = 4). These experiments were performed at least three times with similar results. Statistical significance was calculated by one-way ANOVA with Turkey's multiple comparison test. * *P* < 0.05, ** *P* < 0.01, *** *P* < 0.001, ns (no significant difference).

The phenotype of lymphoma cells is the result of acquired genetic abnormalities and the biological effect of residual lymphocytes, directing the affected site of lymphoma and the composition of the microenvironment. Extensive crosstalk between lymphoma cells and microenvironment cells leads to recruitment and “re-education” of these cells, providing a favorable environment for lymphoma development and progression 18. TAMs, at the core of immunosuppressive cell and cytokine network, have been demonstrated to play a crucial role in tumor immune evasion 10. In the light of the composition of cytokine milieu and surrounding tissular niche, TAMs differentiate into a variety of phenotypic states, but usually be simplified into two categories: classically activated macrophages (M1) and alternatively activated macrophages (M2) respectively 19. Therefore, we tried to verify whether NHL cells directly affect the polarization direction of macrophages. Herein, macrophages were respectively cultured in different conditions followed by polarization phenotype analysis. As shown in **Fig. 1A**, NHL-primed macrophages exhibited an M2-like phenotype, with the expression of CD206 and CD163 similar to that of IL-4-primed macrophages. Besides, exposure of macrophages to NHL cells led to phosphorylation of STAT6, as observed during IL-4 activation (**Fig. 1B**)**.** Meanwhile, IL-4-polarized M2 was found to inhibit the proliferation of CD19-CAR T cells (**Fig. 1C**). To clarify whether NHL-polarized macrophages would exert similar effects, we detected the proliferation ability of CAR T cells under different coculture conditions. Results showed that NHL-polarized macrophages presented a similar effect as IL-4-polarized macrophages in inhibiting the expansion of CAR T cells (**Fig. 1D**). All of the above suggest that the immunosuppressive TME of NHL not only polarizes macrophages into the M2 phenotype, but also inhibits the proliferative activity of CAR T cells through M2.

### CD47 is overexpressed in NHL cells and correlated with adverse prognosis

**Figure 2:**
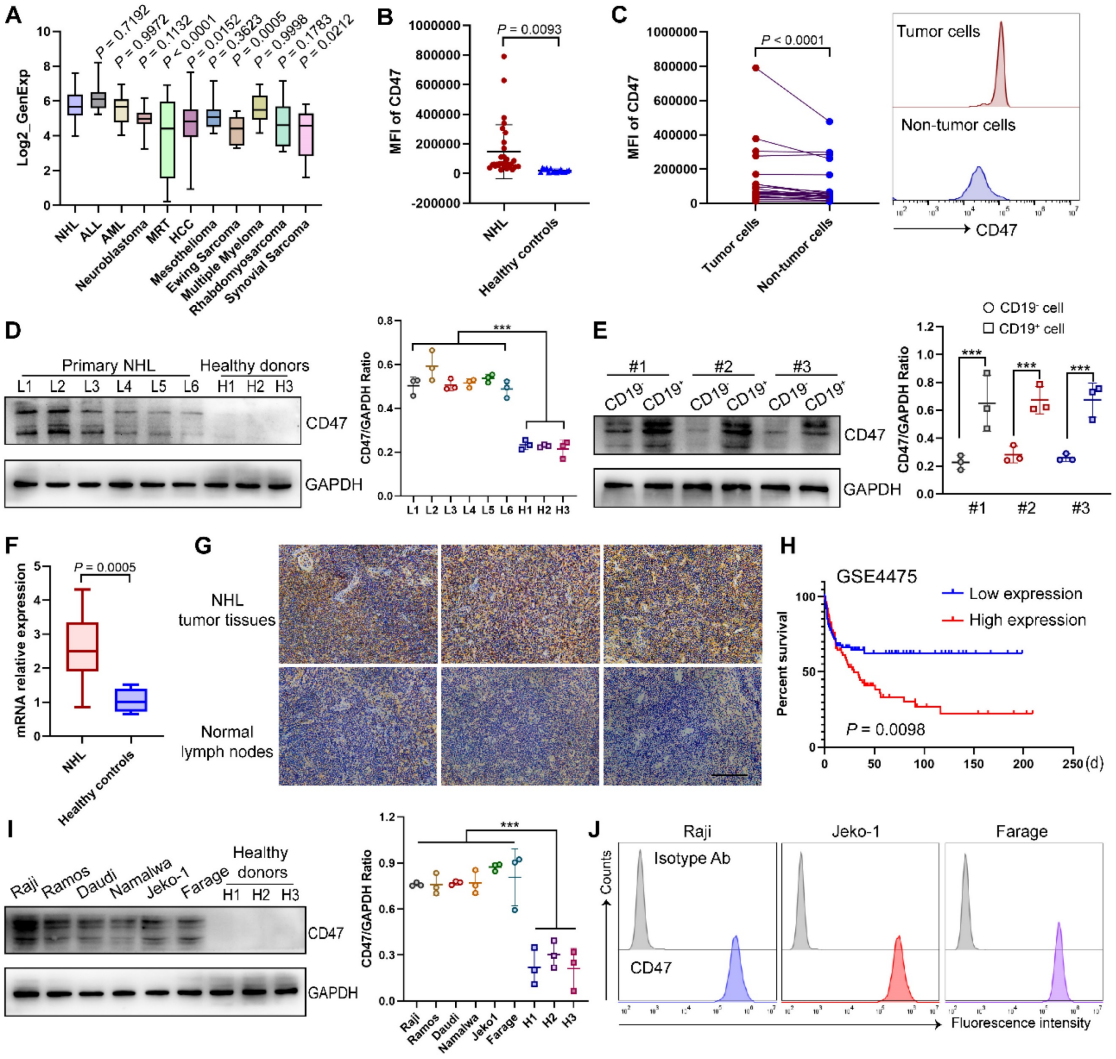
** Identify CD47 as a potent target of NHL. (A)** The expression of CD47 in NHL compared to other indicated tumors, based on data obtained from the CCLE. **(B)** Mean fluorescence intensity (MFI) of CD47 in CD19^+^ tumor cells from NHL BM samples (n = 30) and normal B cells in PB samples from healthy donors (n = 15) detected by flow cytometry analysis. **(C)** Higher CD47 expression in the CD19^+^ fraction compared with that in the CD19^-^ fraction in 25 NHL patients. Statistical chart (left), representative flow cytometry images (right). **(D)** Western blot analysis (left) of CD47 expression in mononuclear cells from NHL patients (n = 6) and healthy donors (n = 3). GAPDH was served as loading control. Statistical analysis plot of the ratio of the IntDen value of CD47 to GAPDH in triplicate experiments (right). **(E)** Western blot analysis (left) of CD47 expression in CD19^+^ tumor cells and CD19^-^ cells from NHL patients (n = 3). GAPDH was served as loading control. Statistical plot (right). **(F)** CD47 mRNA expression in samples from NHL patients (n = 17) and healthy individuals (n = 6) tested by qPCR analysis. **(G)** Representative photomicrographs of IHC staining for CD47 in the NHL tumor tissues (n = 10) and lymph nodes from non-NHL patients (n = 5). Scale bar, 10 μm. **(H)** Integrated analysis of GSE4475 indicating that high CD47 expression is significantly associated with shorter overall survival in NHL patients (low expression, n = 111; high expression, n = 110). **(I)** Western blot analysis (left) of CD47 expression in different NHL cell lines (n = 6) and PB-derived mononuclear cells from healthy donor (n = 3). GAPDH was served as loading control. Statistical plot (right). **(J)** Representative flow cytometry images evincing high CD47 expression in NHL cell lines (Raji, Jeko1, Farage). The experiments in **B-G**, **I**-**J** were performed at least three times with similar results. For **B**-**F**, **I**, data are presented as mean ± standard deviation (SD). For **B** and **F**, data were analyzed by the unpaired Student's t-test. For **C**, Ratio paired t test. For **D-E**, **I**, one-way ANOVA with Turkey's multiple comparison test. For **H**, log-rank tests. * *P* < 0.05, ** *P* < 0.01, *** *P* < 0.001.

Specific or high expression on tumor cells is a prerequisite for being a suitable therapeutic tumor target. Utilizing the CCLE database, we examined the expression pattern of CD47 and found that it was widely expressed in diverse tumors (**Fig. 2A**) 20, 21. To validate CD47 as a target antigen of CAR T cell therapy for NHL, we analyzed the expression intensity of CD47 in clinical samples. As expected, CD47 expression was significantly higher in NHL patients than in healthy controls, and in NHL samples, CD47 expression was also higher in CD19^+^ tumor cells than in non-tumor cells (**Fig. 2B-C**), which were further confirmed by western blotting analysis and qPCR assay (**Fig. 2D-F**). Importantly, immunohistochemical staining of control normal lymph nodes and NHL tumor samples showed a relatively high proportion of CD47^+^ cells in the latter group (**Fig. 2G**). Moreover, survival data in GEO dataset showed that higher CD47 expression of NHL patients was significantly correlated with shorter overall survival (**Fig. 2H**). To define an appropriate human tumor model for further studies, we evaluated several NHL cell lines and found CD47 to be highly expressed by these cells (**Fig. 2I-J**).

### Characterization of CD19-CAR T cells secreting CD47 blocking scFv

**Figure 3:**
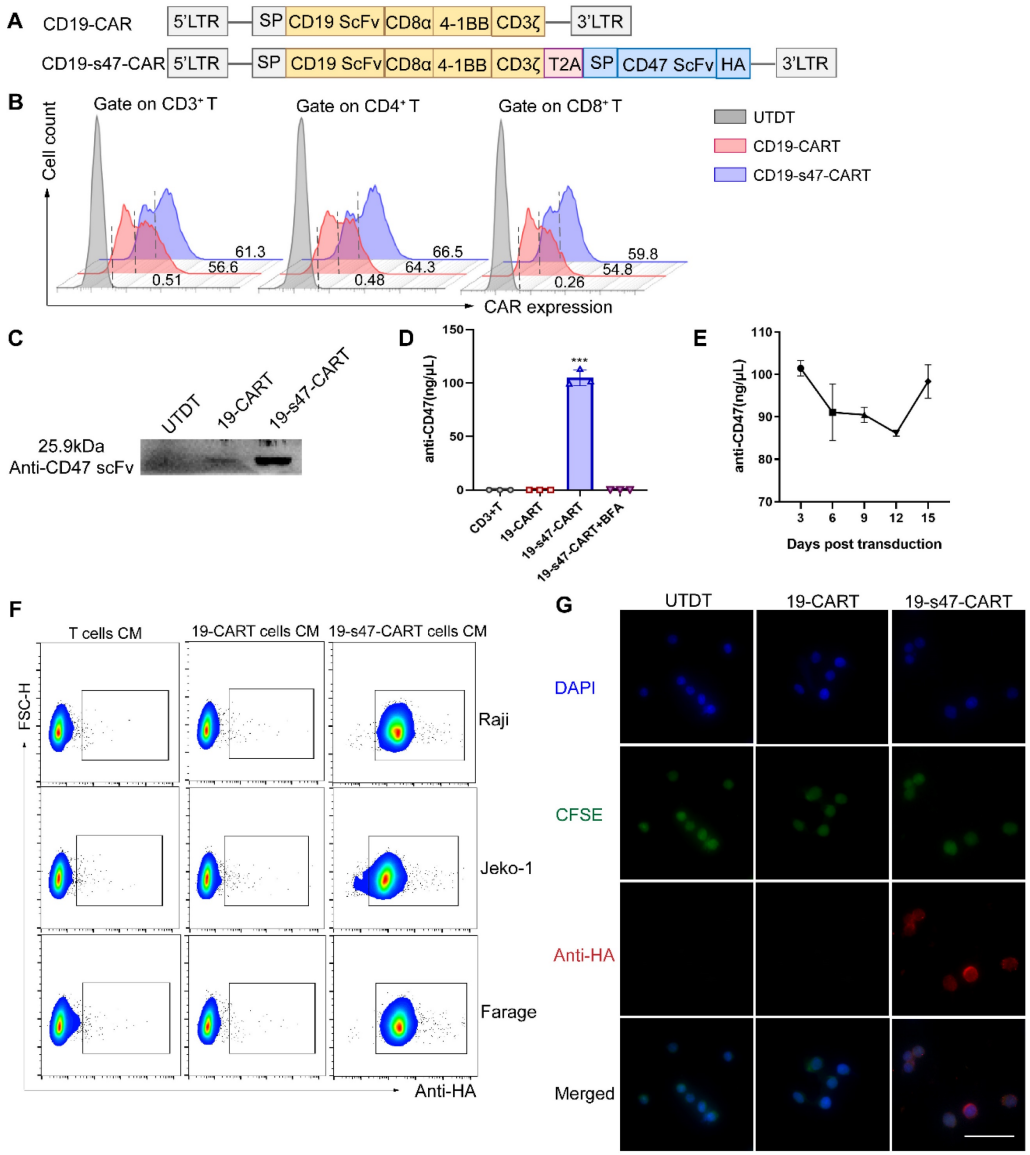
** Construction and characterization of CD19-CAR T and CD19-s47-CAR T cells. (A)** Schematic representation of parental CD19-CAR and anti-CD47 secreting CD19-CAR (CD19-s47-CAR) constructs. CD19-CAR is successively connected by a CD8α signal peptide, an anti-CD19 scFv, a CD8α hinge, a CD8α transmembrane, a 4-1BB intracellular costimulatory domains and an intracellular CD3ζ domain. CD19-s47-CAR is composed of CD19-CAR linked with anti-CD47 scFv, and a HA tag was also included for detection of the secreted scFv. **(B)** Representative flow cytometry plots demonstrating CAR expression in human T cell. A fluorescently labeled CAR-specific antibodies (CARGREEN) was utilized to test the transfection efficiency. Untransduced T cells (UTDT) were used as a control. **(C)** Western blot was performed to analyze the expression of secreted anti-CD47 scFv in the supernatant from T cells. **(D)** Secreted antibodies in supernatants were enriched by HA immunoprecipitation kit and quantified by BCA quantification (n = 3). **(E)** Supernatants of CD19-s47-CAR T cells at different days post viral transfection were collected and quantified as in **D** and the concentration curve was plotted (n = 3). **(F)** Detection of the binding ability of secreted scFv in the culture medium (CM) to NHL cells by flow cytometry. NHL cells were incubated with culture medium from different T cells for 30 min at 37°C and then stained by anti-HA flow antibody. Representative flow charts are exhibited. **(G)** Visualization of anti-CD47 scFv binding to Raji cells by immunofluorescence. Scale bar, 50 μm. These experiments were performed at least three times with similar results.

In view of limitations of existing anti-CD19 CAR T cells, a novel anti-CD47 scFv secreting CD19-CAR T cells was constructed to further uplift the structure and function. A schematic representation of the retroviral vector constructs used in this study is shown in **Fig. 3A**. Human CD3 positive T lymphocytes were separated and transfected with viruses derived from these two vectors. The transfection efficiency of the viruses into T cells were verified at DNA and RNA levels (**Fig. S1A-B**); both of them achieved 50-60% expression rates at protein level (**Fig. 3B**). Western blotting was performed to assess the secretion of the anti-CD47 scFv on the T cell supernatant 5 days after transfection (**Fig. 3C**). The amount of secreted anti-CD47 scFv in the supernatant was also quantified (approximately 100 ng/μL, **Fig. 3D**). During the expansion of CD19-s47-CAR T cells, we collected cell supernatants of different culture days for detection, and found that the secretion of the anti-CD47 was maintained at a relatively stable level (**Fig. 3E**, 85-103ng/μL). The binding activity of anti-CD47 scFv to the surface of NHL cells was also detected by flow cytometry (**Fig. 3F**). Compared with the control medium incubation, the secreted anti-CD47 scFv effectively bound to the surface of target cells, coinciding with the results of immunofluorescence assay (**Fig. 3G, Fig. S1C**).

### Secretion of anti-CD47 antibodies enhances antigen-specific immune responses of CAR T cells

**Figure 4:**
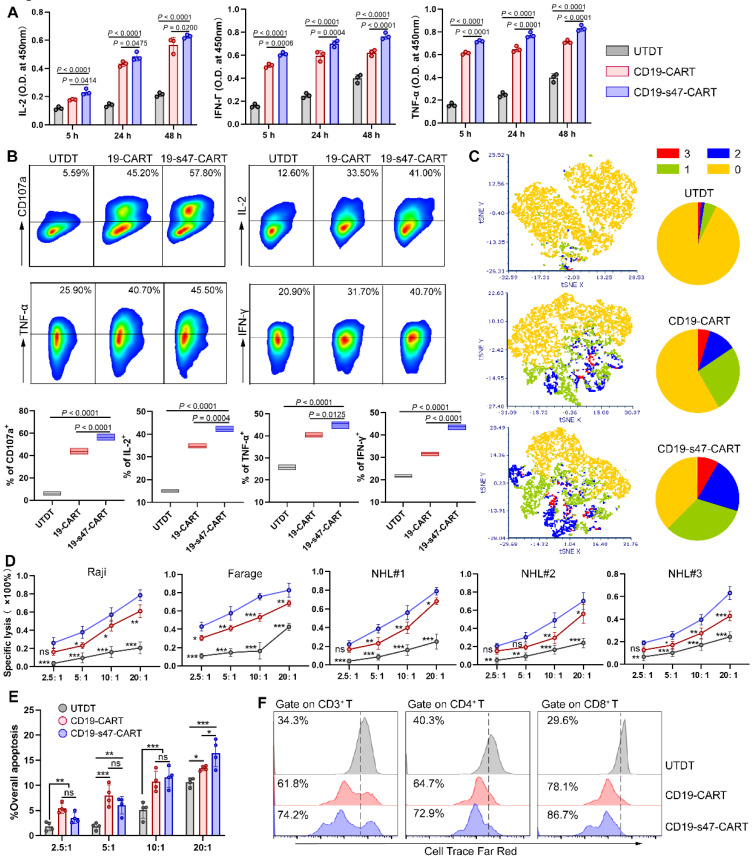
** Detection of antigen-specific immune response of CAR T cells. (A)** CAR T cells were cultured with Farage cells at 37°C for different durations as indicated (E:T=1:1) and then the levels of cytokine secretion (IL-2, IFN-γ and TNF-α) in supernatants were identified by ELISA (n = 3). **(B)** Flow cytometry analyses were conducted to examine degranulation (CD107a, n=5) and cytokine secretion (IL-2, IFN-γ and TNF-α, n=3) of T cells cultured with Farage cells for 4 h. Representative flow cytometry images gating on CD3^+^ cells (upper) and statistical chart (below) are shown. **(C)** Cytokine production was detected via multiple staining followed by flow cytometer analysis. For each sample, 10,000 cells were subjected to tSNE distribution by the FCS Express 7 software. After determining the tSNE x and tSNE y coordinates for each cell, cells were labeled according to polyfunctionality (left). Pie charts summarizing the polyfunctionality assay were also provided (right). 0, 1, 2, 3 are defined as the number of positive cytokine types. **(D)** LDH cytotoxicity assays were carried out to investigate the specific cytotoxicity of UTDT, CD19-CAR T, and CD19-s47-CAR T cells towards NHL cell lines and primary NHL patient samples after 4 h coculture at E:T ratios of 2.5:1, 5:1, 10:1, 20:1 (n = 3). **(E)** Apoptosis assay showing the killing effect of T cells on CFSE-labeled CD34^+^ stem cells (cultured as **D**, n = 4). The apoptotic cells are defined as 7AAD^+^. **(F)** Cell proliferation rates of T cells were explored following antigen-specific stimulation via a 72 h Cell Trace Far Red dilution assay. These experiments were performed at least three times with similar results. All data are presented as the mean ± SD and analyzed by one-way ANOVA with Turkey's multiple comparison test. * *P* < 0.05, ** *P* < 0.01, *** *P* < 0.001, ns (no significant difference).

To further analyze the effector function of CD19-s47-CAR T cells upon antigen stimulation, T cells were cocultured for different durations with NHL cells. Subsequently, the culture supernatants were collected and the secretion of T cell function markers were measured by ELISA, unveiling the upshot that CD19-s47-CAR T cells secreted significantly higher levels of IL-2, IFN-γ and TNF-α compared with the parental CD19-CAR T cells in different culture periods (**Fig. 4A**). Consistent results were obtained applying flow cytometry that CD19-s47-CAR T cells secreted higher proportions of cytokines upon stimulation with tumor antigens (**Fig. 4B, Fig. S2A**). These results suggest that anti-CD47 scFv secretion may induce CAR T cells to produce more abundant cytokines and exert more efficient antitumor effects. To further validate this speculation, we performed multiplex cytokine staining followed by utilizing t-distributed stochastic neighbor embedding (tSNE) and found that CD19-s47-CAR T cells possessed significant polyfunctionality, which has been shown to provide superior protective immunity in various human cancers 22 (**Fig. 4C, Fig. S2B**).

Next, specific tumor lysis activity of engineered T cells was examined by cytotoxicity assay at effector/target (E/T) ratios of 2.5, 5, 10 and 20. Compared with untransduced T cells (UTDT), both CAR T cells mediate significant NHL cell lysis capacity, while CD19-s47-CAR T cells mediated more potent killing function (**Fig. 4D, Fig. S2C**), without significantly killing healthy donor-derived CD34^+^ stem cells compared to parental CD19-CAR T cells (**Fig. 4E**). Apoptosis assay further verified the aforementioned killing effects (**Fig. S2D**). Additionally, although both CAR T cells proliferated faster in response to antigen stimulation compared to UTDT, CD19-s47-CAR T cells exhibited a higher rate of cell division (**Fig. 4F**).

### Secreting anti-CD47 scFv exerts an immunomodulatory effect on T cells

**Figure 5:**
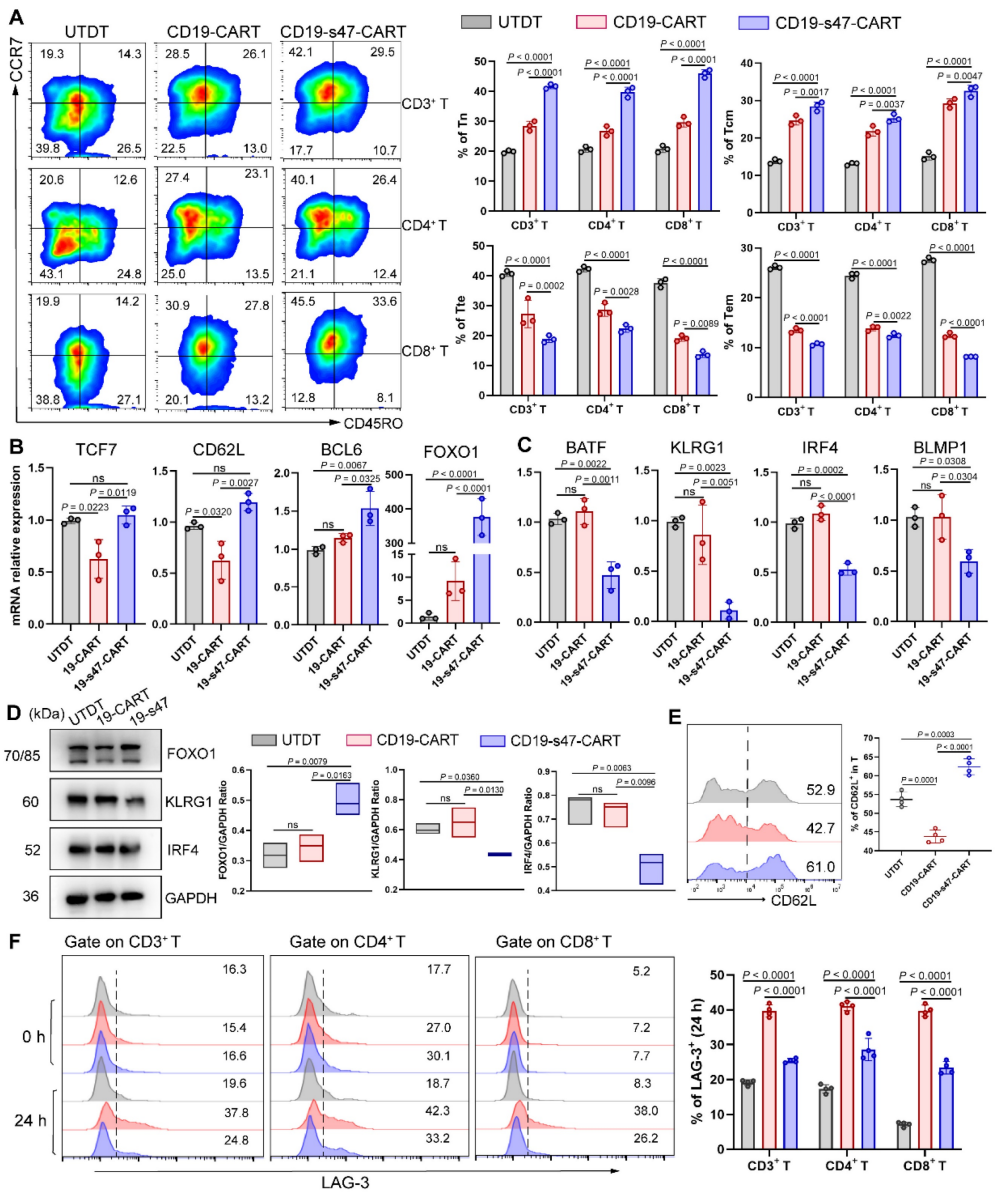
** The immunomodulatory effect created by secreted anti-CD47 antibodies on T cells. (A)** Farage cells were utilized to culture with different T cells at 37°C for 24 h (E:T=1:1). Representative flow images and corresponding quantitative plots exhibiting the proportion of Tn, Tcm, Tte and Tem of CD3^+^, CD4^+^, and CD8^+^ T cells in different groups (n = 3, see Fig. S3A for gating strategy). **(B)** After being cultured as **A**, T cells were separated by CD3 positive selection kit. QPCR analyses were performed to examine the expression level of T cell memory-associated genes (TCF7, CD62L, BCL6 and FOXO1) in groups (n = 3). **(C)** QPCR analyses were performed to examine the expression level of T cell terminal differentiation-related genes (BATF, KLRG1, IRF4 and BLMP1) in groups (n = 3). **(D)** Western blot analyses (left) were conducted to examine the expression level of FOXO1, KLRG1 and IRF4 in groups (n = 3). GAPDH was served as loading control. Statistical analysis plot of the ratio of the IntDen value of proteins to GAPDH in triplicate experiments (right). **(E)** Representative flow images (left) and corresponding quantitative plots (right) exhibiting the positive proportion of CD62L in T cells in different groups (n = 3). **(F)** The LAG-3 expression level of T cells before (0 h) and after (24 h) co-incubation with NHL cells was detected (n = 3). All experiments were performed at least three times with similar results and presented as the mean ± SD. Data were analyzed by one-way ANOVA with Turkey's multiple comparison test.

Poor persistence of CAR T cells is one of the leading causes of recurrence after treatment 23. It has been demonstrated that the proportion of poorly differentiated T cell subsets such as naïve-like T cells (Tn) and central memory T cells (Tcm) in CAR T cells is positively correlated with their *in vivo* expansion ability, duration as well as antitumor effect 24. To investigate whether the secretion of anti-CD47 scFv would favorably influence CAR T cells, we examined cell differentiation of CAR T cells by detecting T cell differentiation markers. Based on CD45RO and CCR7 expression, T cells could be classified into four differentiation subsets: Tn (CD45RO^-^ CCR7^+^), Tcm (CD45RO^+^ CCR7^+^), terminal effector T cells (Tte, CD45RO^-^CCR7^-^) and effector memory T cells (Tem, CD45RO^+^ CCR7^-^) (**Fig. S3A**) 25. An increased percentage of T cells with Tn and Tcm phenotypes, and a decreased proportion of T cells with Tte and Tem phenotypes were observed in both the CD4^+^ and CD8^+^ T cell subsets of CD19-s47-CAR T cells compared to other groups (**Fig. 5A**). Besides, we performed qPCR analysis and found that TCF7, CD62L, BCL6 and FOXO1 (associated with T cell memory) were upregulated, while BATF, KLRG1, IRF4 and BLMP1 (associated with terminal differentiation) were downregulated in CD19-s47-CAR T cells (**Fig. 5B-C**). The differential expression of FOXO1, CD62L, KLRG1 and IRF4 in groups was further validated at protein level (**Fig. 5D-E**).

Cell surface immune checkpoint inhibitory molecules (ICIs), including PD-1, LAG-3, TIM-3, and CTLA-4, play critical roles in inducing inhibitory signals and limiting antitumor efficacy of CAR T cell therapy 26. To evaluate whether the expression of ICIs is regulated by CAR stimulation, we measured the expression of them on CAR-engineered T cells and found that ICI expression was upregulated in both CAR T groups (**Fig. 5F, Fig. S3B**). In comparison, CAR T cells secreting anti-CD47 scFv expressed a lower level of ICIs than CD19-CAR T cells upon Raji stimulation, while ICIs were expressed at a similar level in all groups without antigen-specific stimulation.

### Impact of anti-CD47 scFv secretion on phagocytosis and polarization of macrophages

**Figure 6:**
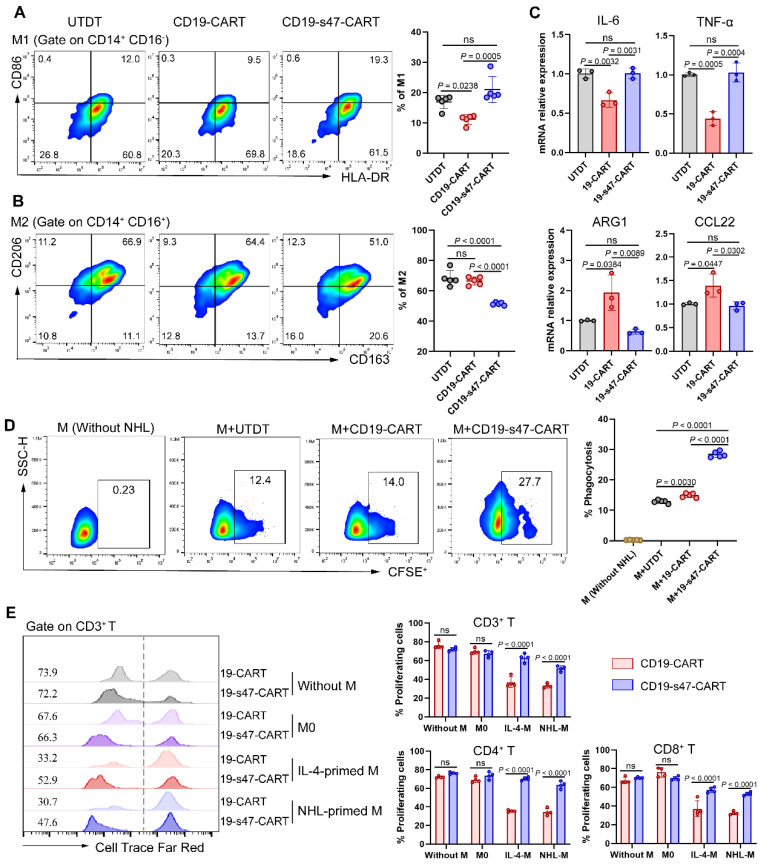
** Secretion of anti-CD47 scFv by CAR T cells modulated the functions of macrophages. (A)**Following incubated with Farage and different T cells for 24 h at a ratio of 1:1:1, macrophage polarization was detected by flow cytometry analysis. The percentage of M1 positive cells for each individual were shown (n = 5, see Fig. S3C for gating strategy). **(B)** The percentage of M2 positive cells for each individual were shown (n = 5). **(C)** Macrophages in **A** were separated by CD14 positive selection kit for qPCR analyses to determine the expression level of IL-6 and TNF-α (M1-associated genes), as well as ARG1 and CCL22 (M2-associated genes) under different culture conditions (n = 3). **(D)** Macrophage phagocytosis assay was performed by cocultured CFSE-labeled Farage cells with macrophages at the presence of different T cells at a ratio of 2:1:1 (NHL to macrophages to T cells) for 3 h. Macrophages were identified by human anti-CD14 flow antibody and the percentage of CFSE^+^ macrophages was characterized as phagocytosis rate (n = 5). **(E)** Cell Trace Far Red-labeled CAR T cells were cultured with macrophages and Farage cells at a ratio of 1:1:1 for 72 h. Representative flow cytometry images (left) and corresponding statistical charts (right) confirmed that NHL-polarized macrophages inhibited proliferation of CD19-CAR T cells, exerting effects similar to IL-4-polarized macrophages, whereas this inhibition effect could be attenuated at the presence of secreted anti-CD47 antibodies (n = 4). All experiments were performed at least three times with similar results and presented as the mean ± SD. Data were analyzed by one-way ANOVA with Turkey's multiple comparison test. For **E**, unpaired Student's t-test.

We previously verified that NHL cells promote macrophages to polarize into an M2 phenotype. Herein, to determine whether secretion of anti-CD47 scFv exerts a regulatory effect on polarization direction of macrophages, we examined the polarization marker expression on macrophages in coculture system composed of NHL cells, CAR T cells, and macrophages 27. Compared to other groups, proportion of M1 was slightly upregulated and proportion of M2 was markedly downregulated in the CD19-s47-CAR T cell group (**Fig. 6A-B, Fig. S3C**). Analogous effects were further confirmed by qPCR assay, which showed that M1-associated genes IL-6 and TNF-α were upregulated, and M2-associated genes ARG1 and CCL22 were significantly downregulated in macrophages cocultured with CD19-s47-CAR T cells (**Fig. 6C**). These results suggest that secreted anti-CD47 antibody effectively regulate macrophage polarization in TME, attenuating their potency to polarize into M2 phenotype.

Given that overexpression of CD47 by tumor cells to circumvent phagocytosis of macrophages is one of the means to induce immune escape 11, we analyzed whether anti-CD47 scFv secretion made a difference to phagocytic efficiency of macrophages against tumor cells. Results showed that CD19-CAR T cells engineered to secret anti-CD47 antibodies could enhance phagocytosis of macrophages compared with the conventional ones (**Fig. 6D**), suggesting that anti-CD47 scFv effectively slackens off the inhibitory impact of macrophage phagocytosis caused by CD47 overexpression in tumor cells.

Above we mentioned that suppressive TME of NHL promotes M2 polarization thereby inhibiting proliferation of CD19-CAR T cells. To further detect whether CD19-s47-CAR T cells were resistant to M2 inhibition, we cocultured CFSE-labeled CAR T cells with M0, IL-4-polarized macrophages, and NHL-polarized macrophages respectively to perform proliferation assay. In contrast, CD19-s47-CAR T cells were more resistant to the proliferation inhibition effect of M2 than parental CAR T cells (**Fig. 6E**). Above results imply secretion of anti-CD47 ameliorates suppressive TME of NHL to some extent.

### CAR T cells secreted anti-CD47 scFv exerts comparable immunomodulatory effects as CD47 antibody Hu5F9

**Figure 7:**
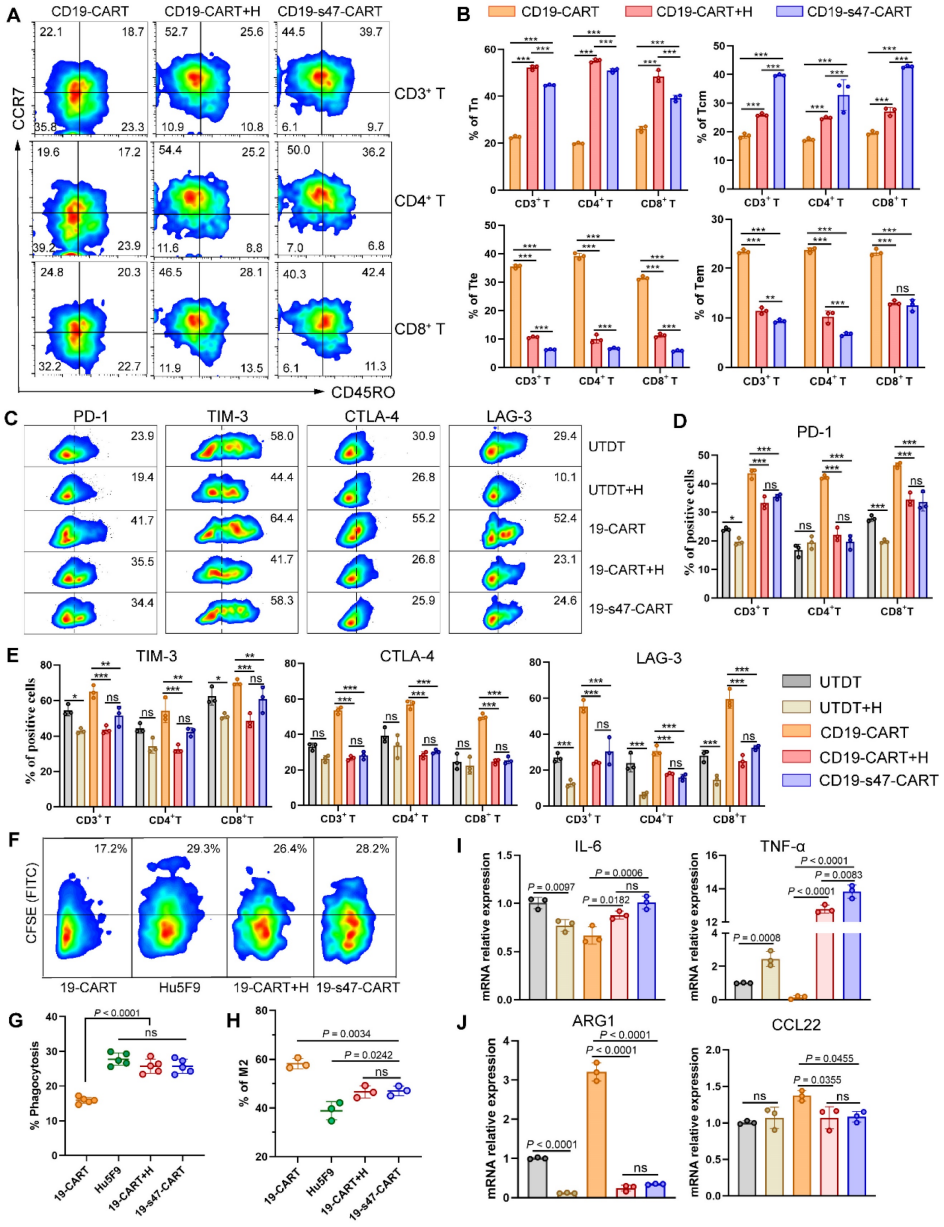
** Comparison between secreted anti-CD47 scFv and Hu5F9 in immunocyte regulation. (A)** T cell differentiation was analyzed based on expression levels of CCR7 and CD45RO to detect proportions of Tn, Tcm, Tte and Tem. T cells were incubated with Raji cells at an E/T ratio of 1:1 for 24 h with or without Hu5F9 (200 ng/mL). Representative flow cytometry images were furnished. **(B)** Corresponding statistical charts of **A** are displayed (n = 3). **(C)** Percentage of PD-1-, TIM-3-, CTLA-4-, and LAG-3- positive cells among different T cells incubated with Raji at a E/T ratio of 1:1 for 24 h with or without Hu5F9 (200 ng/mL). **(D-E)** Statistical charts of **C** recapitulating the ICIs expression levels in different group (n = 3). **(F)** The impact of secreted anti-CD47 antibody or Hu5F9 (200 ng/mL) on macrophage phagocytosis was determined by analyzing the percentage of CFSE^+^ cells in CD14^+^ cells. **(G)** Statistical charts of **F** are shown (n = 5). **(H)** Percentage of CD163^+^ CD206^+^ macrophages were inspected by flow cytometry to compare the influence of secreted anti-CD47 scFv or Hu5F9 (200 ng/mL) on macrophage polarization (n = 3). **(I-J)** QPCR analyses were performed to determine mRNA expression levels of M1-associated genes (IL-6, TNF-α, **I**) and M2-associated genes (ARG1, CCL22, **J**) in macrophages cultured with Raji and various T cells for 24 h at a ratio of 1:1:1 in the presence or absence of Hu5F9 (200 ng/mL, n = 3). All experiments were performed at least three times with similar results and presented as the mean ± SD. One-way ANOVA with Turkey's multiple comparison test was applied for statistical analyses.

As a promising tumor target, multiple clinical trials of CD47 antibodies are in progress and have achieved considerable antitumor effects 15, 28. Herein, a classical CD47 antibody Hu5F9 was applied to compare the immunomodulation function between anti-CD47 scFv secreting CAR T cells and antibody. After being cocultured with NHL cells for 24 h with or without Hu5F9, increased proportions of Tn and Tcm were respectively observed in the combined group and the dual-target CAR T group (**Fig. 7A-B**). Moreover, the presence of anti-CD47 antibody reduced the expression of ICIs on T cells (**Fig. 7C-E**), increased the expression of T cell memory-related genes, and reduced the expression of terminal differentiation-related genes (**Fig. S4A-D**) to some extent, no matter in the form of exocrine or drug administration. However, Hu5F9 neither enhanced the competence of CAR T cells to secrete CD107a upon antigen stimulation as CD19-s47-CAR T cells did (**Fig. S4E**), nor strengthened the polyfunctionality of T cells (**Fig. S4F**).

As for the impact on macrophage function, targeting CD47 augmented the phagocytic efficiency of macrophages on CFSE-labeled NHL cells in antibodies present groups when opposed to CD19-CAR T cell monotherapy group (**Fig. 7F-G**). In addition, both the appearance modes of anti-CD47 antibodies restrained macrophage differentiation to immunosuppressive phenotypes (**Fig. 7H-J**). As a consequence, except that CD19-s47-CAR T cell possesses a more potent antigen-stimulated immune response, it also manifests analogous immunoregulatory effect as Hu5F9 administration.

### Anti-CD47 engineered CAR T cells exhibit enhanced* in vivo* antitumor reactivity

**Figure 8:**
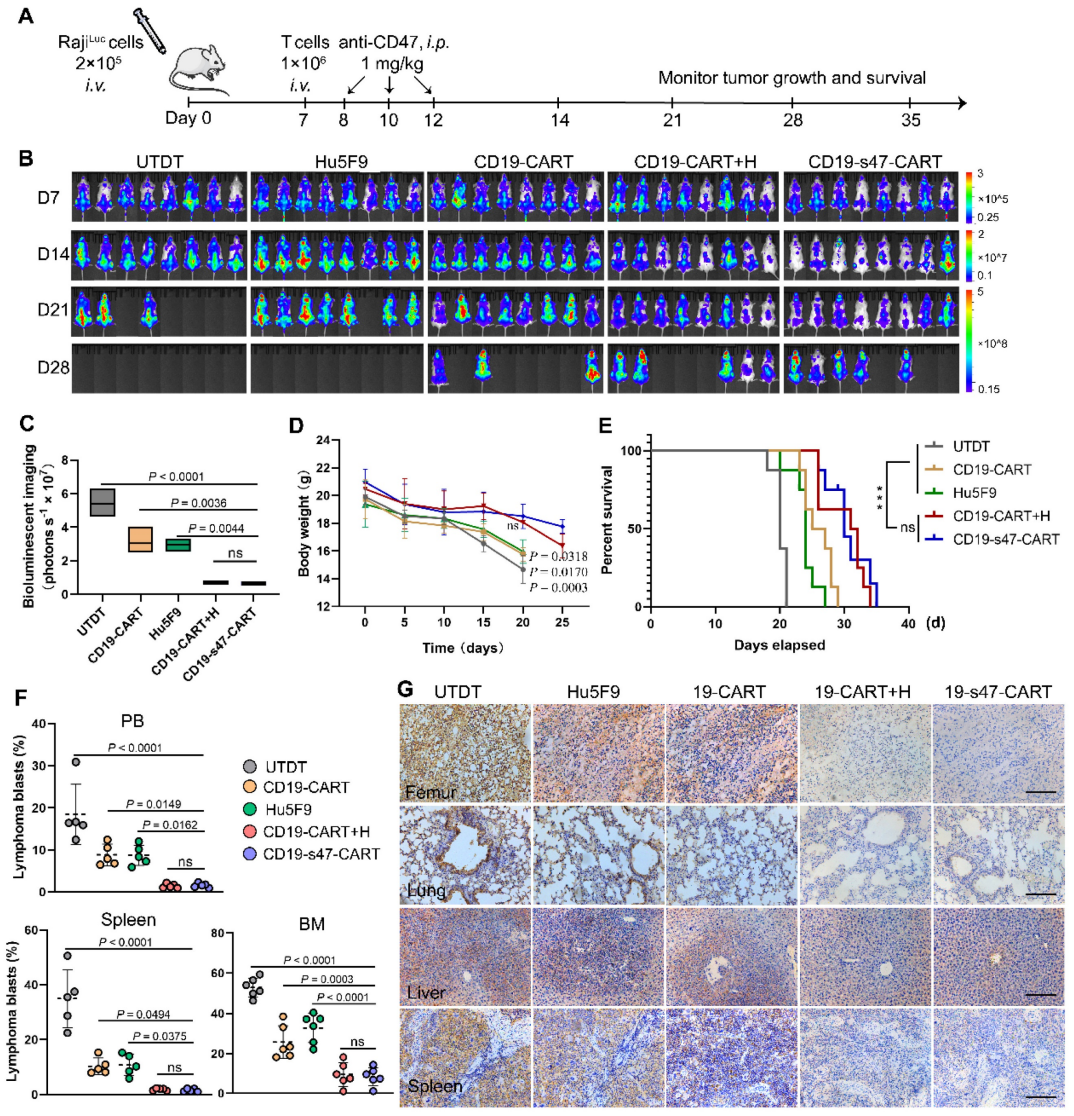
** Improved therapeutic effects of CD19-s47-CAR T cells against pre-established NHL tumor models. (A)** Protocol scheme of *in vivo* experiment for tumor challenge, T cell adoptive transfer and Hu5F9 administration. B-NDG mice were injected with 2 × 10^5^ luciferase-expression Raji cells via tail vein. On day 7, different T cells were adoptively transferred through intravenous injection. One day after T cell infusion, Hu5F9 was administered via intraperitoneal injection (*i.p.*) at a dose of 1 mg/kg on the indicated date. Mice were observed daily for status and body weight, and IVIS imaging was performed weekly to monitor tumor progression. **(B)** Representative images of tumor burden obtained by bioluminescent imaging following D-luciferin substrate administration (150 mg/kg, *i.p.*) at each analysis time point (day 7, 14, 21, and 28, n = 8). The floating bars on the right extended from the minimum to maximum. **(C)** Quantitative statistics of bioluminescent imaging indicating tumor burden (day 21). Bioluminescent imaging values are represented as photons s^-1^ cm^-2^ sr^-1^ in regions of interest with the entire body of each mouse. **(D)** Body weight change curves of mice receiving different treatments. *P* values were provided for each group compared to the CD19-s47-CAR T cell group. **(E)** Kaplan-Meier survival curves showed that the survival of NHL mice was significantly improved in the anti-CD47 secreting CAR T cells group and the combination group. **(F)** Scatter plots exhibiting tumor blasts (GFP^+^) in PB, spleen, and BM from the indicated cohorts at the experimental endpoint of each mouse (day 21, n = 5). **(G)** Representative photomicrographs of IHC staining for human CD19 showing tumor infiltration of femur, lung, liver, and spleen in different treatment conditions. Scale bar, 10 μm. Data in **C**, **D**, and **F** are presented as mean ± SD and analyzed by One-way ANOVA with Turkey's multiple comparison test. For **E**, statistical significance was calculated by two-sided log-rank Mantel-Cox tests.

To evaluate the *in vivo* antitumor efficacy of CD19-s47-CAR T cells, we adoptively transferred 1 × 10^6^ CAR T cells into xenograft mouse models established by intravenous injection of luciferase-expressing Raji cells into B-NDG mice. Schematic outline of experimental procedure for animal study was shown in **Fig. 8A**. Tumor progression was monitored by IVIS Spectrum following D-luciferin substrate administration. Evident organ infiltration by NHL cells can be observed on day 7. In comparison with the UTDT, Hu5F9, or CD19-CAR T cell groups, CD19-s47-CAR T cells significantly delayed tumor development, while there was no significant difference compared with the combined treatment group (**Fig. 8B-C**). Correspondingly, much more stable body weights were spotted in the CD19-s47-CAR T group and the combined treatment group (**Fig. 8D**). Such a potent therapeutic effect thus led to a much more prolonged survival time (**Fig. 8E**). Albeit CD19-CAR T cell as well as anti-Hu5F9 monotherapy showed antitumor activity to some extent, CD19-s47-CAR T cells further decreased tumor burden and prolonged the survival time of mice compared with monotherapy groups, achieving comparable effect with the combined treatment group. More evidences were provided by demonstrable suppression and reduced infiltration of blasts in various tissues (**Fig. 8F-G, Fig. S5A**).

H&E staining of sections from major organs also testified the role of secreted anti-CD47 scFv in delaying tumor cell infiltration and maintaining normal organ morphology and histology (**Fig. S5C**). Moreover, routine blood tests for mice after treatment were carried out to verify whether the modified double-target CAR T cells were safer than antibody application. Although no statistical difference was observed, the WBC count in the CD19-s47-CAR T cell group seems to be less affected (**Fig. S5B**). Conspicuously, a significant decrease in hemoglobin emerged in groups with antibody application, but not in the CD19-s47-CAR T cell group, implying the potential safety of this novel CAR T cell.

**Figure 9:**
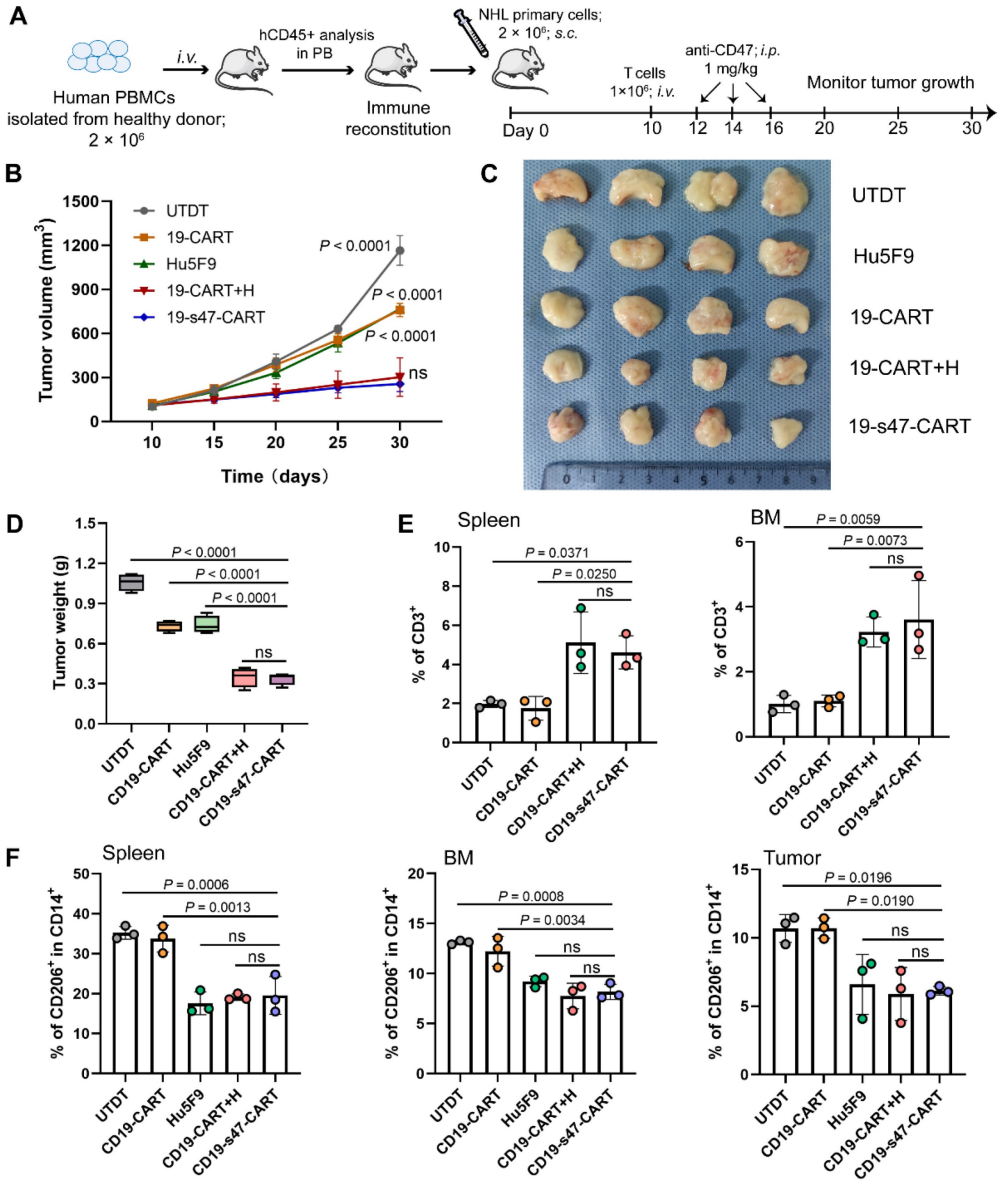
** Improved anti-tumor and immunomodulation effects of CD19-s47-CAR T cells in humanized mouse models. (A)** Schematic representation of the experimental plan used to evaluate anti-NHL activity and immunomodulation effect *in vivo* using PBMCs-PDX mice derived from primary NHL cells. **(B)** Primary NHL cells (P4) were implanted subcutaneously into B-NDG mice, and the tumor volume was recorded. **(C)** 30 days after subcutaneous inoculation of NHL cells, mice were sacrificed, tumor tissues were removed, then tumors were photographed. **(D)** Tumor tissues in **C** were weighed. **(E)** Flow cytometry analyses showing human CD3^+^ cells in spleen and BM at the experimental endpoint of each mouse (n = 3). **(F)** Frequency of M2-like (CD206^+^) TAMs in monocytes (CD14^+^) in spleen, BM and tumor from mice (n = 3). Data in **B**, **D**, and **E-F** are presented as mean ± SD and analyzed by One-way ANOVA with Turkey's multiple comparison test.

Humanized PBMCs-PDX mouse models were also established to further verify the anti-tumor properties and immunomodulatory effects of the double-target CAR T cells (**Fig. 9A**). As demonstrated, the novel CAR T cells and the combination treatment groups significantly delayed tumor progression (**Fig. 9B**). On Day 30, all tumors were separated and individually weighed. Tumors in CD19-s47-CAR T cell and combination group mice were significantly lighter than those in other group mice (**Fig. 9C-D**). Besides, we also examined the proportion of several immune cells in tissues of mice to interrogate the *in vivo* immunomodulatory effects of secreted anti-CD47 antibodies. Results showed that the presence of the antibodies contributed to enhancing CAR T cell persistence (**Fig. 9E**), and abating proportion of M2 immunosuppressive cells (**Fig. 9F**), which were instrumental in immunotherapy outcomes.

## Discussion

In this research, we generated a novel anti-CD47 scFv secreting CD19-CAR T cell and verified its efficacy, safety and immunomodulatory effects on TME. The secreted scFv efficiently bound to CD47, reversing the upregulated ICIs and functional depletion of T cells spawned by immunosuppressive TME of NHL, promoting T cells differentiate into immunologic memory phenotypes, thereby enhancing antitumor efficacy. Utilizing xenograft mouse models, we also demonstrated that CD19-s47-CAR T cells further enhance *in vivo* antitumor effects, extend the continuity of T cells, and prolong the overall survival of mice when compared with parental CD19-CAR T cells. Whilst CD47 blockade by constitutively secreted anti-CD47 scFv also blocked anti-macrophage phagocytosis of tumor cells and diminished polarization of macrophages to the inhibitory phenotype, which significantly ameliorated TME.

One possibility for the high recurrence rate of CAR T cell immunotherapy is the profound penetration of immunosuppressive factors in the NHL TME, consisting of inhibitory cytokines and numerous immunosuppressive cells such as TAMs, inhibits the activity of CAR T cells 29. Our research indicated that exposure to NHL cells activated STAT6 signal transduction pathway related to macrophage polarization and polarized macrophages towards an IL-4-like phenotype, which inhibited CAR T cell proliferation. As a consequence, it is a starting point worth exploring to enhance the efficacy of CAR T cells by attenuating inhibitory components in TME.

Previous studies have proved that ICI expression on CAR T cells could be upregulated under continuous stimulation of various immunosuppressive factors and tumor antigens, which renders T cell functionally exhausted, characterized by decreased killing function, gradual loss of proliferative capacity, and finally apoptosis 30. Proportion of poorly differentiated T cell subsets such as Tn and Tcm in CAR T cells also plays a part in *in vivo* expansion competence and duration 24, 31. To overcome existing limitations mentioned above, several studies have been conducted to engineer CAR T cell structure, among which there are ways to reform with ICIs targets like PD-1, effectively ameliorating TME, but little difference was found between the parental and reformed CAR T cells in terms of cytolytic activity 32, 33. We wondered whether a target not only exerting immunomodulatory function, but also promoting cytotoxicity towards tumor cells could be found or not. The recently emerging immune checkpoint CD47 has been shown to be vital in circumventing immune surveillance of various tumors, and CD47 blockade enhances phagocytosis of myeloid cells to tumor cells, promoting the antigen cross presentation of antigen presenting cells, thus initiating T cell function and activating adaptive immune response.

Based on this logic strategy, we designed CD19-s47-CAR T cells, with which CAR T cells secreted scFv that efficiently bind to CD47, improving antitumor efficacy and polishing up the TME. The presence of anti-CD47 scFv rescued T cell inhibition mediated by TME of NHL, restrained the differentiation of T cells to depletion phenotype, and induced a gene expression mode that was enriched with memory differentiation-related genes. Additionally, anti-CD47 scFv secretion resulted in more functional CD19-s47-CAR T cells with higher proliferation capacity, characterized by potent cytokine production and granulation. Of note, the elevated cytokine secreting ability also featured as polyfunctionality compared with parental CD19-CAR T cells, which was rarely explored in the homologous design of CAR T cells. Besides, CD47 antibodies secreted by the CAR T cells have also been demonstrated to reduce inhibitory polarization and fortify phagocytosis of macrophages, conducive to improve immunotherapy outcomes.

Considering CAR T cells traffic to and expand at tumor site, local delivery of anti-CD47 scFv could potentially abate the toxicity of systemic CD47 blockade, which was manifested in our *in vivo* experiments. Moreover, the smaller size of scFv facilitated tissue penetration and distribution. Our work also suggested that both the dual-target CAR T cell group and the combined treatment group fulfilled an analogous role in adjusting immune reaction of T cells and macrophages. Nevertheless, CD19-s47-CAR T cells exerted a stronger immune response stimulated by specific antigen, registered as more efficient degranulation function and cytokine production with polyfunctionality, which was not available in the group additionally supplemented with CD47 antibody. This phenomenon may be associated with the activation of intracellular pathways in anti-CD47 scFv secreting CAR T cells responding to antigen stimulation, and specific mechanism remains to be explored in the next step.

To sum up, the novel engineered CAR T cells evince alleviated hypofunction, enhanced proliferation and ameliorated immunotherapy outcomes, implying that self-secreting anti-CD47 could be another promising method to augment the competence of CAR T cells in tumors with heavy microenvironmental infiltration. For future researches, other than CD19, this approach could be applied to CAR T cells of targets such as HER2 or CLDN18.2 that have been proved to be effective but limited to the inhibitory TME.

## Supplementary Material

Supplementary figures and tables.
