# Supplementary material for: Enhanced tumor immunotherapy by polyfunctional CD19-CAR T cells engineered to secrete anti-CD47 single-chain variable fragment
Source: Int J Biol Sci. 2023 Sep 18;19(15):4948–66. doi: 10.7150/ijbs.86632 (PMC10539696; doi:10.7150/ijbs.86632)
Supplement: Supplementary file 1 — Supplementary figures and tables. [file ijbsv19p4948s1.pdf]

Supplementary Materials for

Enhanced tumor immunotherapy by polyfunctional CD19-CART cells engineered to secrete anti-CD47 single-chain variable fragment

Yingqi Qiu[1*], Peiyun Liao[1*], Hao Wang[1*], Jianyu Chen[1], Yuxing Hu[1], Rong Hu[1], Honghao Zhang[1], Zhongwei Li[1], Manxiong Cao[1], Yulu Yang[1], Suwan Wu[1], Meifang Li[1], Xiaoling Xie[1#], Yuhua Li[1, 2#]

[1]Department of Hematology, Zhujiang Hospital, Southern Medical University, Guangzhou, Guangdong, 510280, P. R. China

[2]Bioland Laboratory (Guangzhou Regenerative Medicine and Health Guangdong Laboratory), Guangzhou, Guangdong, 510005, P. R. China

[*] These authors contribute equally to this work.

[#] Co-corresponding authors:

Yuhua Li, Department of Hematology, Zhujiang Hospital, Southern Medical University, Guangzhou, Guangdong, China, 510280; Phone and fax numbers: 86-020-62782316; E-mail: liyuhua1974@outlook.com

Xiaoling Xie, Department of Hematology, Zhujiang Hospital, Southern Medical University, Guangzhou, Guangdong, China, 510280; Phone and fax numbers: 86-020-62782317; E-mail: xxl413@smu.edu.cn

Supplementary Figures

Fig. S1

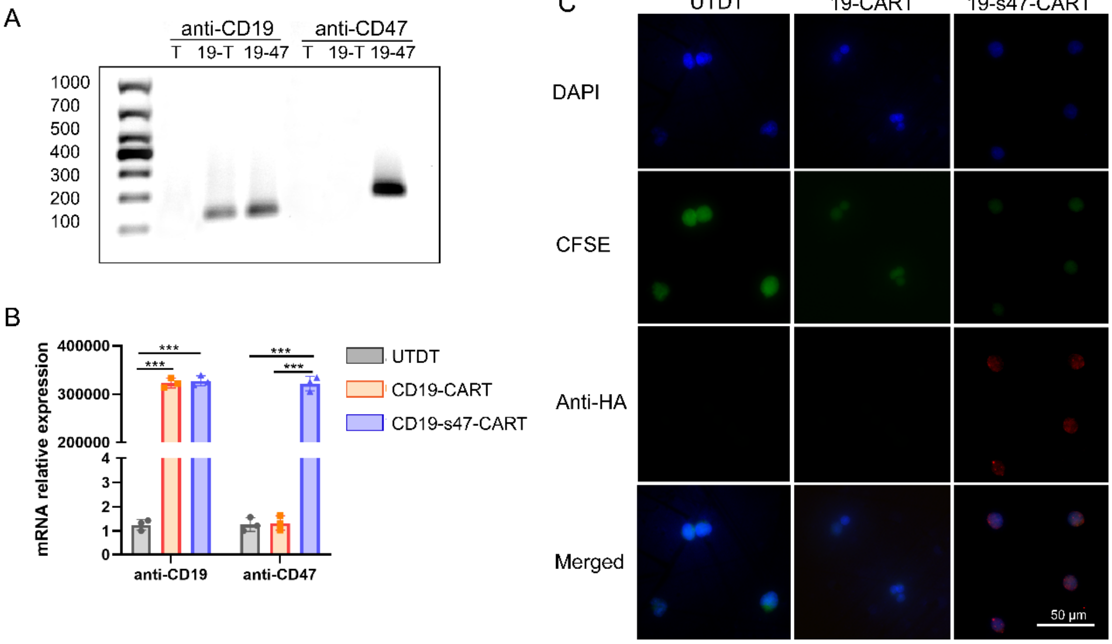

Fig. S1. Human T cells were genetically modified to secrete CD47-blocking scFv.

A. DNA of T cells was extracted then amplified by PCR followed by DNA electrophoresis to verify the expression of CAR fragments.

B. QPCR was performed to validate CAR expression at RNA level (n = 3).

C. Visualization of anti-CD47 scFv binding to Farage cells by immunofluorescence.

All experiments were performed at least three times with similar results. Data in **B** were presented as the mean ± SD and analyzed by one-way ANOVA with Turkey's multiple comparison test. * $P < 0.05$, ** $P < 0.01$, *** $P < 0.001$, ns (no significant difference).

Fig. S2

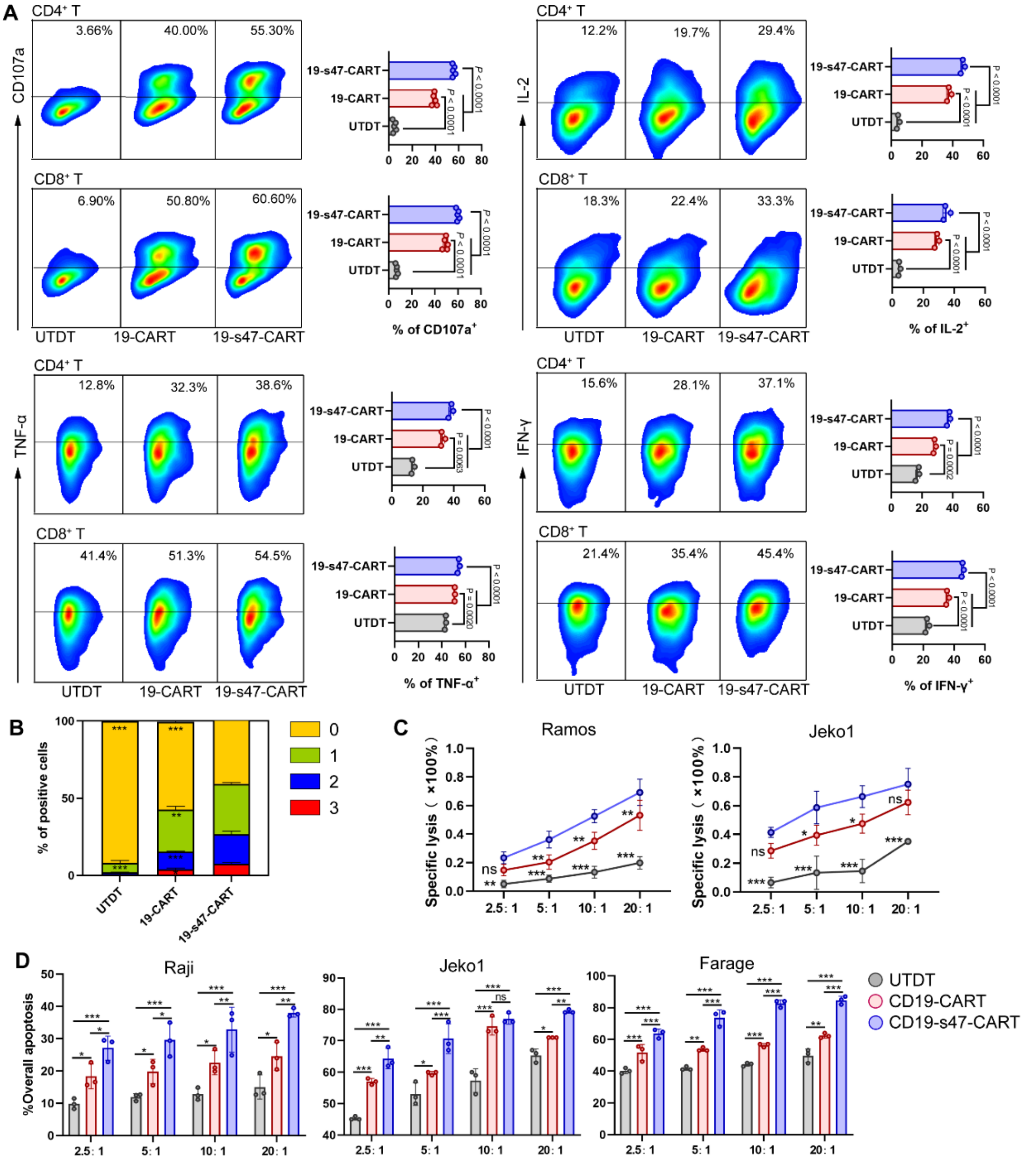

Fig. S2. CD19-s47-CART cells mounted a more robust immune response upon antigen stimulation.

A. Representative flow images and corresponding quantitative plots showing the proportion of positive cells for CD107a (n = 5), IL-2 (n = 3), IFN-γ (n = 3) and TNF-α (n = 3) in $CD8^+$ as well as $CD4^+$ T cells in different groups after stimulated with Farage cells for 5 h.

B. Stacked bar chart of Fig. 4C display different degrees of polyfunctionality of T cells in different groups (n = 3). *P* value of CD19-s47-CART cells group compared with other two groups have been indicated in the figure.

C. LDH cytotoxicity assays indicating the specific cytotoxicity of diverse T cells to NHL cell lines (Ramos, Jeko1, n = 3). *P* value of CD19-s47-CART cells group compared with other two groups have been indicated in the figures.

D. Apoptosis assay showing the killing effect of T cells on CFSE-labeled NHL cells at E:T ratios of 2.5:1, 5:1, 10:1, 20:1 (n = 3).

All experiments were performed at least three times with similar results. Data are presented as the mean ± SD and analyzed by one-way ANOVA with Turkey's multiple comparison test. * $P < 0.05$, ** $P < 0.01$, *** $P < 0.001$, ns (no significant difference).

Fig. S3

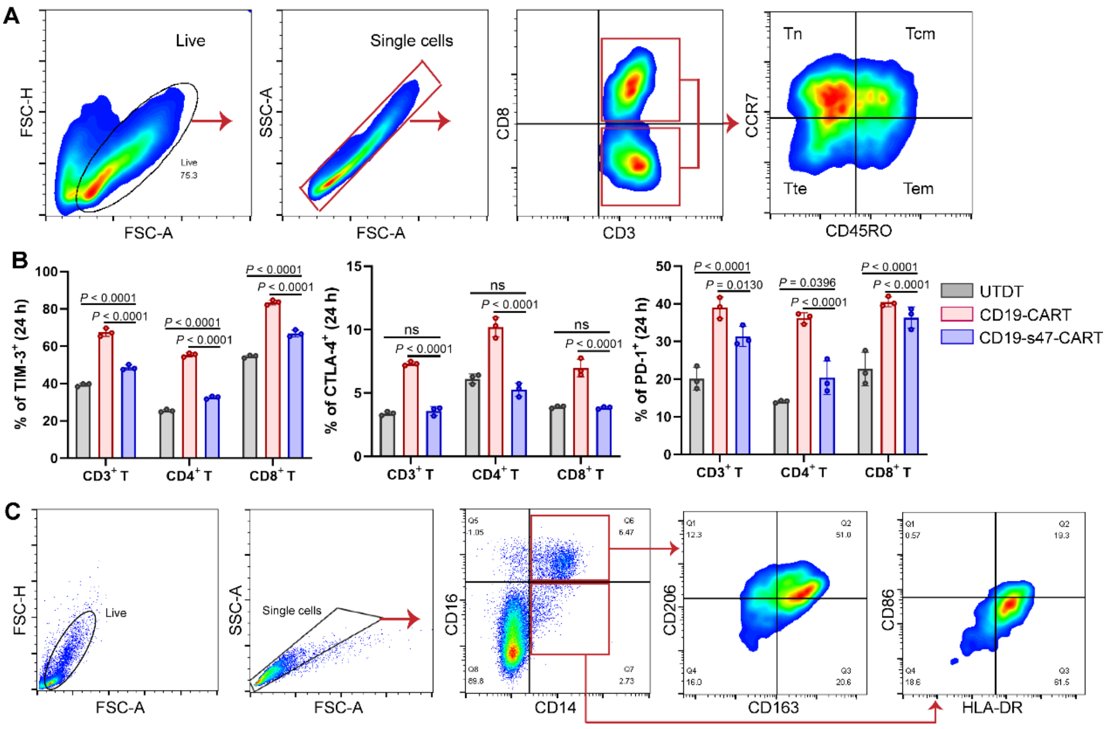

Fig. S3. Secreting anti-CD47 possessed immunomodulatory effect on TME.

A. Gating strategy of T cell differentiation analysis. $CD3^+$, $CD4^+$, and $CD8^+$ T cells are classified into four differentiation subsets based on CD45RO and CCR7 expression: Tn ($CD45RO^-$ $CCR7^+$), Tcm ($CD45RO^+$ $CCR7^+$), Tte ($CD45RO^-CCR7^-$) and Tem ($CD45RO^+$ $CCR7^-$).

B. Statistical charts revealing ICIs (TIM-3, CTLA-4, and PD-1) expression on T cells stimulated by antigen (n = 3). Data are presented as the mean ± SD and analyzed by one-way ANOVA with Turkey's multiple comparison test.

C. Gating strategy of macrophage polarization phenotyping. Following exclusion of debris and doublets, cells were divided into $CD14^+$ $CD16^+$ and $CD14^+$ $CD16^-$ subsets, and then further analyzed for the polarization marker expression of M1 ($HLA\text{-}DR^+$ $CD86^+$) and M2 ($CD163^+$ and $CD206^+$), respectively.

Fig. S4

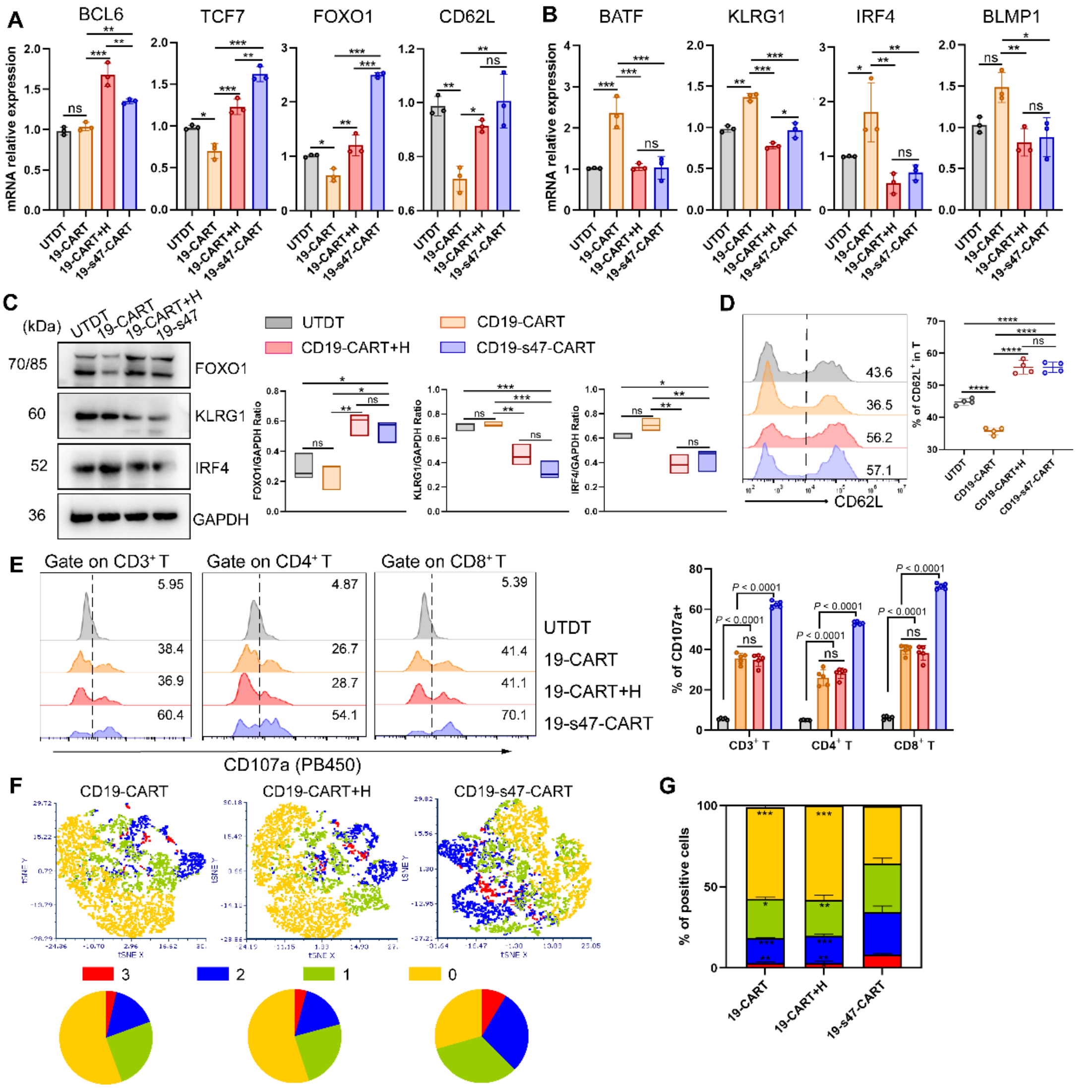

Fig. S4. Secreted anti-CD47 scFv elaborated similar immunomodulatory effect as Hu5F9.

A. Following being incubated with Raji cells at a E/T ratio of 1:1 for 24 h with or without Hu5F9 (200 ng/mL), T cells were separated by CD3 positive selection kit for qPCR analyses to determine mRNA expression levels of T cell memory-associated genes BCL6, TCF7, FOXO1, and CD62L (n = 3).

B. MRNA expression levels of T cell terminal differentiation-related genes (BATF, KLRG1, IRF4, and BLMP1) in different treatment conditions (n = 3).

C. Western blot analyses (left) were conducted to examine the expression level of FOXO1, KLRG1 and IRF4 in groups (n = 3). GAPDH was served as loading control. Statistical analysis plot of the ratio of the IntDen value of proteins to GAPDH in triplicate experiments (right).

D. Representative flow images (left) and corresponding quantitative plots (right) exhibiting the positive proportion of CD62L in T cells in different groups (n = 3).

E. Representative flow cytometry images (left) and corresponding statistical charts (right) showing T cell degranulation after cultured with Raji cells at a E/T ratio of 1:1 for 4 h. In combined treatment group, Hu5F9 was supplemented at a concentration of 200 ng/mL (n = 5).

F. Cytokine secretion in different treatments were detected via multiple staining followed by flow cytometer analysis. Graphs unfolding tSNE distribution (top) and corresponding pie charts (bottom) are shown.

G. Stacked bar graph of **F** was exhibited (n = 3).

All experiments were performed three times with similar results and presented as the mean ± SD. One-way ANOVA with Turkey’s multiple comparison test was applied for statistical analyses. * $P < 0.05$, ** $P < 0.01$, *** $P < 0.001$, ns (no significant difference).

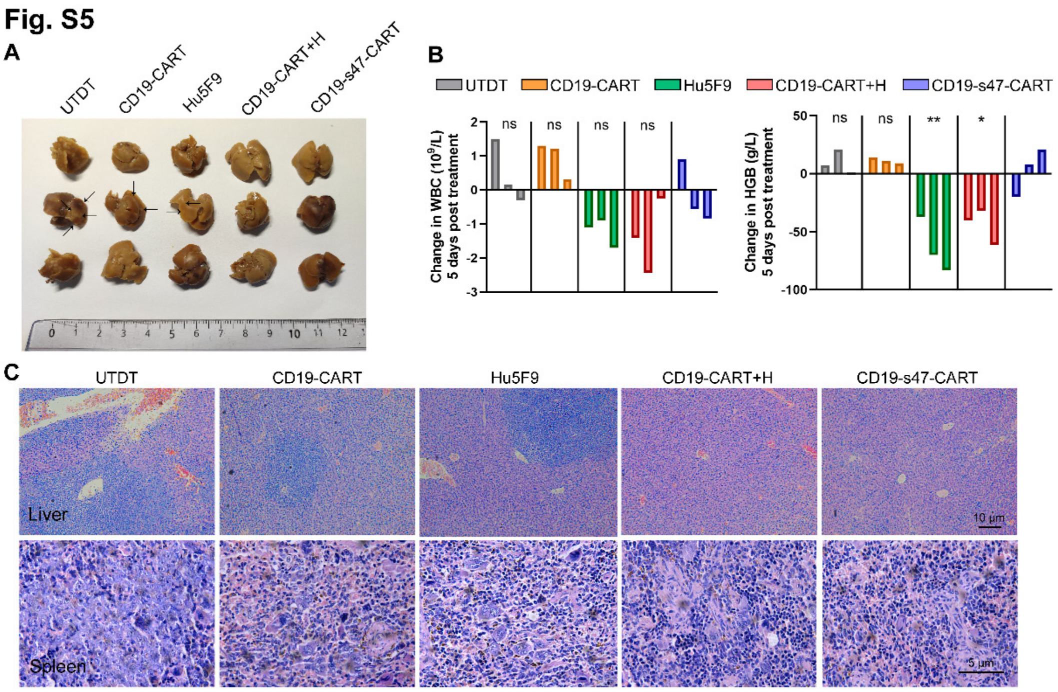

Fig. S5. ***In vivo*** **safety and immunomodulatory effect of CD47 antibodies in tumor-bearing mice.**

A. Representative morphology images of livers from mice receiving different treatment.

B. The waterfall plots unveiling changes in absolute WBC counts (10^9/L) and hemoglobin (HGB) (g/dL) on day 5 post treatment in groups (n = 3).

C. H&E-stained liver and spleen sections from mice in groups.

Data are presented as the mean ± SD and analyzed by One-way ANOVA with Turkey's multiple comparison test.

Supplementary Tables

Table S1. Clinical characteristics of NHL patients, related to Fig. 1B.

Patients	Gender	Age	% Blasts	Status	Cytogenetics
1	Male	64	7.51	Newly diagnosed	-
2	Male	67	82.97	Newly diagnosed	FR1-JH, FR2-JH, FR3-JH, DH-JH, VK-JK
3	Male	43	23.4	Relapsed	TET2, BRCA2, SMC3, CREBBP
4	Male	62	57.95	Newly diagnosed	-
5	Male	77	25.46	Newly diagnosed	IGH/CCND1
6	Male	68	85	Newly diagnosed	IGH/CCND1
7	Male	38	25.94	Newly diagnosed	IGH/MYC
8	Male	80	15.06	Relapsed	IGH/CCND1, TP53
9	Male	61	10.77	Newly diagnosed	-
10	Male	64	44.38	Newly diagnosed	IGH/CCND1, TP53
11	Male	65	26.33	Newly diagnosed	KMT2D, B2M
12	Female	53	10.97	Relapsed	-
13	Male	68	34.04	Relapsed	NOTCH1, FBXW7
14	Male	77	15.58	Relapsed	IGH/MYC
15	Male	54	75.57	Relapsed	IGH/MYC
16	Male	52	44.81	Newly diagnosed	-
17	Male	61	73.54	Relapsed	-

18	Male	59	56.72	Newly diagnosed	-
19	Male	77	36.86	Newly diagnosed	ATM, NOTCH2, IGH/CCND, IGH/MYC, IGH/BCL2
20	Female	66	46.93	Newly diagnosed	BCL2, BCL6
21	Male	71	72.62	Newly diagnosed	TP53, KMT2D, MYD88, ASXL3, BTK, IL7R, KMT2A, MUC16, SETD2
22	Female	47	94.10	Relapsed	NOTCH1, IGLL5, MGA
23	Male	12	72.4	Newly diagnosed	FLT3-ITD, NF1, FAT3
24	Male	56	45.00	Relapsed	BCL2, BIRC3, SGK1
25	Male	47	34.55	Relapsed	-
26	Female	51	22.89	Newly diagnosed	BRAF, TET2
27	Male	47	31.06	Relapsed	-
28	Female	63	21.08	Newly diagnosed	-
29	Male	68	44.53	Newly diagnosed	IGHV, ATM, SF3B1, TRAF3, CREBBP
30	Male	63	24.23	Newly diagnosed	-

Table S2. Amino acid sequences of CAR scFv.

	Amino acid sequence
human anti-CD19 scFv	DIQMTQTTSSLSASLGDRVTISCRASQDISKYLNWYQ QKPDGTVKLLIYHTSRLHSGVPSRFSGSGSGTDYSLTI SNLEQEDIATYFCQQGNTLPYTFGGGTKLEITGGGGS GGGGSGGGGSEVKLQESGPGLVAPSQSLSVTCTVSGV SLPDYGVSWIRQPPRKGLEWLGVIWGSETTYYNSALK SRLTIIKDNSKSQVFLKMNSLQTDDTAIYYCAKHYYY GGSYAMDYWGQGTSVTVSS
human anti-CD47 scFv	DVVMTQSPLSLPVTPGEPASISCRSSQSIVYSNGNTYL GWYLQKPGQSPKLLIYKVSNRFSGVPDRFSGSGSGTD FTLKISRVEAEDVGVYHCFQGSHVPYTFGGGTKVEIK GGGGSGGGGSGGGGSQVQLVQSGAEVKKPGASVKV SCKASGYTFTNYNMHWVRQAPGQGLEWIGTIYPGND DTSYNQKFKDKATLTADKSTSTAYMELSSLRSEDTAV YYCARGGYRAMDYWGQGTLVTVSS

Table S3. List of antibodies.

Antibodies	Source	Identifier
PE/Cyanine7 anti-human CD163	Biolegend	Cat#326514
PE anti-human CD206	Biolegend	Cat#321106
Pacific Blue anti-human HLA-DR	Biolegend	Cat#307624
APC anti-human CD86	Biolegend	Cat#305412
FITC anti-human CD14	Biolegend	Cat#325604
PerCP/Cyanine5.5 anti-human CD16	Biolegend	Cat#302028
PE anti-human CD47	Biolegend	Cat#323108
PerCP/Cyanine5.5 anti-human CD45	Biolegend	Cat#304032
APC anti-human CD19	Biolegend	Cat#302212
PE Anti-HA.11 Epiitope Tag	Biolegend	Cat#901517
APC anti-human CD3	Biolegend	Cat#300412
PE anti-human CD4	Biolegend	Cat#300508
PerCP/Cyanine5.5 CD8	Biolegend	Cat#300912
Pacific Blue anti-human CD107a	Biolegend	Cat#328624
APC anti-human IL-2	eBioscience	Cat#17-7029-82
PE/Cyanine7 anti-human IFN-γ	Biolegend	Cat#502527
Brilliant Violet 421™ anti-human TNF-α	Biolegend	Cat#502931
APC anti-human CCR7	Biolegend	Cat#353214
PE anti-human CD45RO	Biolegend	Cat#304206
APC anti-human PD-1	Biolegend	Cat#329908

PE anti-human TIM-3	Biolegend	Cat#345005
PE anti-human CTLA-4	Biolegend	Cat#349905
PE anti-human LAG-3	Biolegend	Cat#369205
Human TruStain FcX™	Biolegend	Cat#422301
PE anti-mouse CD206	Biolegend	Cat#141705
APC/Fire^{TM} 750 anti-mouse CD11b	Biolegend	Cat#101261
PE anti-mouse Ly6C/G	Biolegend	Cat#127607
Anti-STAT6 (phosphor Y641)	Abcam	Cat#ab263947
Anti-STAT6	Abcam	Cat#ab32108
Anti-CD47 antibody	Abcam	Cat#ab218810
HA-tag (4G3) monoclonal antibody	Bioworld	Cat#AP0005M
CD19 polyclonal antibody	Bioworld	Cat#BS6980

Table S4. Sequences of the primers used for qPCR.

Gene name	Primer sequence (5' to 3')
CD47	F: AGCTCTAGCACAATTACTTGGAC
	R: AAGTGATTCCTTTCACGTCT
TCF7	F: ATGGTCACAAGCACAAAGCTC
	R: ACTTAACCTATTCCATTCCCCTT
CD62L	F: CCCCAGACCTTTTATCCAC
	R: GCAGGATTTATTCAAATGCAA
BCL6	F: GTTTCTTTTGTATGTAAATGTGC
	R: CAACTCTGCCATATATTCCT
FOXO1	F: ACTGCTGGATATATGCTACCAA
	R: TGGTCTGTTCGCATAAACCAC
BATF	F: CATGCCTCACAGCTCCGACA
	R: CTTGATCTCCTTGCGTAGAGCC
KLRG1	F: GCTCTTCACACGTAATGCAA
	R: TGCCTTATCGAATTGACTGCT
IRF4	F: CTTCTTAATTCTCCAAGCGGAT
	R: ATTCAGCTCCACTGTTAAAGCA
BLMP1	F: AATTTCCCCAAAGCATAGGTG
	R: CTGAGCCTATCTACCTCGAA
IL-6	F: ACTCACCTCTTCAGAACGAATTG
	R: CCATCTTTGGAAGGTTCAGGTTG

TNF-α	F: CCTGGTATGAGCCCATCTATC<br>R: CGAAGTGGTGGTCTTGTTGC
ARG1	F: GTGGAAACTTGCATGGACAAC<br>R: AATCCTGGCACATCGGGAATC
CCL22	F: ATCGCCTACAGACTGCACTC<br>R: GACGGTAACGGACGTAATCAC
GAPDH	F: GGAGCGAGATCCCTCCAAAAT<br>R: GGCTGTTGTCATACTTCTCATGG

Table S5. Patient information for IHC analysis.

Patients	Disease	Gender	Age
1	Diffuse large B-cell lymphoma	Male	63
2	Diffuse large B-cell lymphoma	Male	32
3	Follicular lymphoma	Female	86
4	Mantle cell lymphoma	Male	66
5	Diffuse large B-cell lymphoma	Male	47
6	Mantle cell lymphoma	Male	55
7	Mantle cell lymphoma	Male	66
8	Diffuse large B-cell lymphoma	Female	82
9	Follicular lymphoma	Female	48
10	Diffuse large B-cell lymphoma	Male	63
11	Chronic lymphadenitis	Male	76
12	Reactive hyperplasia of lymph nodes	Female	68
13	Reactive hyperplasia of lymph nodes	Female	21
14	Reactive hyperplasia of lymph nodes	Female	20
15	Chronic lymphadenitis	Male	45
